# Functional Analysis of Ligand‐Gated Chloride Channels in a Cnidarian Sheds Light on the Evolution of Inhibitory Signaling

**DOI:** 10.1002/advs.202515481

**Published:** 2026-05-01

**Authors:** Abhilasha Ojha, Linda Kloss, Juan D. Montenegro, Simone Albani, Audrey Ortega‐Ramírez, Mihaela Raycheva, Sylvia Joussen, Sabrina Kaul, Michèle Bachmann, Lisa Huf, Günther Schmalzing, Alison G. Cole, Ulrich Technau, Stefan Gründer

**Affiliations:** ^1^ Institute of Physiology RWTH Aachen University Aachen Germany; ^2^ Department of Neurosciences and Developmental Biology Faculty of Life Sciences University of Vienna Vienna Austria; ^3^ Institute for Neuroscience and Medicine (INM‐9) Computational Biomedicine Forschungszentrum Jülich Jülich Germany; ^4^ Institute of Clinical Pharmacology RWTH Aachen University Aachen Germany

**Keywords:** cnidaria, cys‐loop receptors, GABA, GABA_A_ receptors, glutamate‐gated ion channels, ligand‐gated ion channels, Nematostella

## Abstract

γ‐aminobutyric acid (GABA) is the predominant inhibitory transmitter in the vertebrate nervous system. Fast inhibitory signaling is mediated by type A GABA receptors (GABA_A_Rs). While GABA is also present in plants and prokaryotes, it is unknown when it was first used for fast neuronal transmission. Cnidaria represent a sister group to all Bilateria and possess a variety of putative GABA_A_Rs, none of which has been functionally characterized. In this study, we surveyed putative inhibitory ion channel receptors from four different cnidarians. Phylogenetic analysis reveals a surprising phylogenetic complexity of these receptors. While the majority form a cnidarian‐specific radiation, others cluster with bilaterian receptors. We functionally analyze seven putative *Nematostella* GABA_A_Rs of the cnidarian radiation and find that none is activated by GABA or glycine, whereas three are activated by glutamate. Using site‐directed mutagenesis, we identify a lysine residue in the canonical ligand‐binding pocket that is important for activation by glutamate. Our results identify a group of inhibitory ion channel receptors in Cnidaria that use glutamate as a ligand. Moreover, they suggest that inhibitory ion channel receptors in Cnidaria massively diversified, which may have been instrumental in the evolution of complex behaviors and sensory processing by the cnidarian nervous system.

## Graphical abstract

We uncover a large variety of putative inhibitory ligand‐gated ion channels (LGICs) in the phylum Cnidaria, the sister group to all bilaterian animals. Phylogenetic analysis suggests a complex evolutionary history of inhibitory LGICs with diverse neurotransmitter ligands. We show that a subgroup of these cnidarian LGICs is activated by glutamate, an excitatory transmitter in vertebrates.

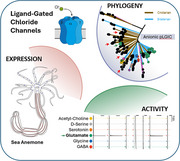

## 1 Introduction

**FIGURE 1:**
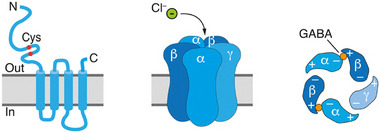
Schematic representation of GABA_A_Rs. Left, cartoon illustrating the transmembrane topology of a single subunit. The characteristic Cys‐loop is indicated. Middle, cartoon illustrating the pentameric structure. In mammals, typical GABA_A_Rs have a 2α2βγ stoichiometry. Right, top‐view cartoon, illustrating the binding of two GABA molecules at the β+/α− interfaces.

The balance between excitatory and inhibitory signaling is a hallmark of nervous systems. Fast inhibitory neurotransmission rapidly reduces neuronal activity and is mediated by ligand‐gated ion channels (LGICs) that are selective to chloride. The major inhibitory neurotransmitter in the vertebrate nervous system is γ‐aminobutyric acid (GABA). LGICs opened by GABA belong to the family of Cys‐loop receptors or pentameric LGICs (pLGICs) (Figure 1). pLGICs comprise anion‐selective and cation‐selective members. Cation‐selective members include nicotinic acetylcholine receptors (nAChRs) and serotonin 5‐HT_3_ receptors (5‐HT_3_Rs). The anion‐selective members include GABA_A_ receptors (GABA_A_Rs), glycine receptors (GlyRs), and, in invertebrates, chloride channels gated by glutamate (GluCls) [1, 2, 3], serotonin (MOD‐1) [4] and other biogenic amines [5], acetylcholine (ACC, LGC‐46) [6], or histamine [7]. Both GlyRs and GluCls share the unique feature of a second Cys‐loop between the signature Cys‐loop and the first transmembrane helix M1 [1, 8]. GABA_A_Rs are typically heteropentamers that mediate both synaptic (phasic) and tonic inhibition [9, 10].

Although neurotransmitter signaling through pLGICs is widespread among bilaterians, its evolutionary origin is less clear. Sponges and placozoans do not have nervous systems, whereas in comb jellies (Ctenophora), which are potentially the first diverging animal lineage [11], pLGICs have not been identified [12]. By contrast, in Cnidaria, the sister group to Bilateria, which diverged about 600 Myr ago, candidates for GABA_A_R can be detected in the genome [13], yet there is no assessment of the functional properties of putative GABA_A_Rs in a cnidarian and specific GABA_A_R‐expressing cell types have not been identified. A major model organism among cnidarians is the sea anemone *Nematostella vectensis* [14], which has a diffuse nerve net in both cell layers [15, 16]. Single‐cell transcriptome atlases have revealed that *Nematostella* has about 25 distinct neuronal cell types [17, 18]. Neurogenesis is driven by a conserved set of transcription factors and signaling pathways, including SoxC, SoxB2, Achaete‐scute and Notch [17, 19, 20, 21, 22]. This suggests that the neurons are homologous to bilaterian neurons. In addition to neurons, there are also other related neural cell types, such as secretory (gland) cells and cnidocytes, the stinging cells [17, 23, 24, 25], and muscle of both ectodermal and mesodermal origin [17, 18, 26]. The nervous system is thought to regulate muscle contraction and the discharge of cnidocytes. While neuropeptides regulate a variety of behaviors in cnidarians [27] and use ionotropic receptors [28], it is currently unclear which other neurotransmitters might be involved.

Here, we report a large family of putative GABA_A_Rs and GlyRs from *Nematostella vectensis*, most of which belong to a cnidarian‐specific radiation of the GABA_A_ subfamily. We cloned and characterized seven pLGICs from this cnidarian‐specific radiation and found that none responded to GABA or glycine, but three were activated by glutamate. We identified a unique lysine residue in the ligand‐binding pocket, which is essential for glutamate sensitivity. Our study is the first to functionally characterize recombinant pLGICs from a cnidarian species and indicates that Cnidaria use glutamate and probably other transmitters besides GABA and glycine for fast inhibitory transmission via pLGICs. Our results lay the foundation for a better understanding of the evolution of fast inhibitory signaling in Cnidaria and in animal nervous systems in general.

## 2 Results

### 2.1 Most Putative GABA_A_Rs in *Nematostella* Belong to a Cnidarian‐Specific Radiation

We identified 39 pLGICs with homology to the GABA_A_R/GlyR superfamily in the newly annotated transcriptome that accompanies the chromosome‐level genome assembly of the anthozoan *Nematostella vectensis* [29], 14 in the medusozoan *Hydra vulgaris* (strain AEP) [30], 21 in the medusozoan *Aurelia coerulea*, and 20 in the anthozoan *Stylophora pistillata*. In contrast, we could not identify putative pLGICs in the genomes of the ctenophores *Mnemiopsis leidyi* and *Bolinopsis infundibulum*, confirming a previous report [12]. We also surveyed the genomes of four sponges (*Oscarella*, *Sycon*, *Amphimedon*, and *Corticium*) and could identify 7 pLGICs in the genome of only one of them, *Corticium candelabrum*. Either the other sponges lost pLGICs or *C. candelabrum* received pLGICs by lateral gene transfer. Because of these uncertainties and highly inconsistent clustering in the phylogenetic analyses, pLGICs from sponges were not further analyzed in this study.

**FIGURE 2:**
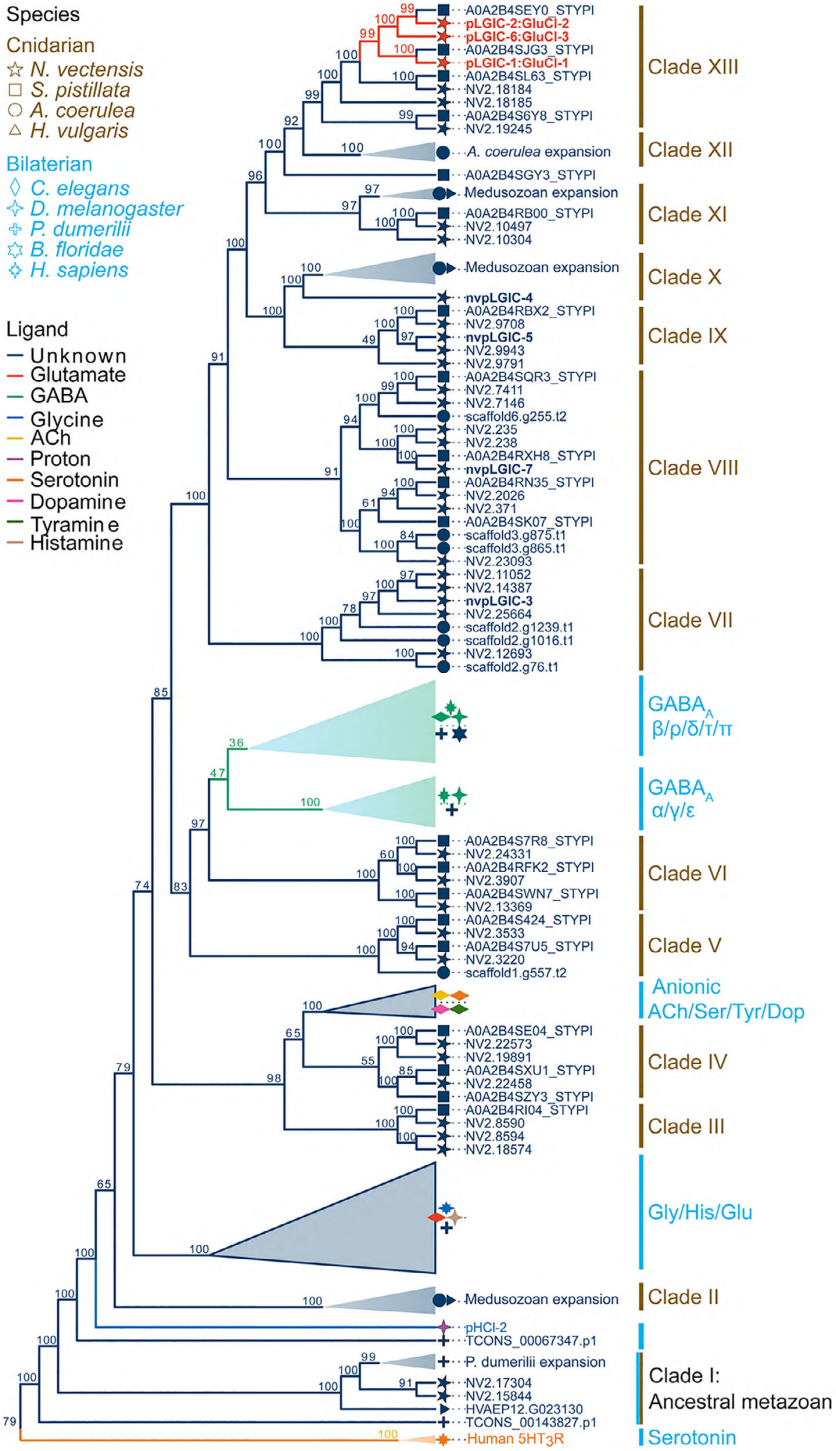
Phylogenetic analysis of cnidarian GABA_A_Rs/GlyRs. Maximum likelihood analysis of pLGIC genes in the cnidarians *N. vectensis*, *S. pistillata*, *A. coerulea, H. vulgaris*, and the bilaterians *C. elegans*, *D. melanogaster*, *P. dumerilii*, *B. floridae*, and *H. sapiens*. Cnidarian clades are highlighted on the right in brown and bilaterian clades in blue. Cloned receptors of this study are shown in bold. Support (bootstrap) values for individual nodes are indicated. The tree was rooted with mammalian 5‐HT_3_Rs as an outgroup. For non‐annotated proteins, labels of receptors from the various species were taken from the genomic annotation to facilitate their identification in the database: receptors from *S. pistillata* start with “SO”, those from *N. vectensis* with “NV”, from *A. coerulea* with “scaffold”, from *H. vulgaris* with “HVAEP”, and from *P. dumerilii* with “TCONS”.

We used the cnidarian pLGICs and performed a thorough phylogenetic analysis with pLGICs from five different bilaterian species, including one annelid, two ecdysozoans, one cephalochordate, and one vertebrate, using mammalian 5‐HT_3_Rs as an outgroup (Figure 2; Figure S1). The phylogenetic analysis revealed that the majority of cnidarian pLGICs belong to a cnidarian‐specific radiation of putative GABA_A_Rs, namely 26 of the 39 *Nematostella* receptors, 10 of the 14 *Hydra* receptors, 15 of the 21 *Aurelia* receptors, and 11 of the 20 *Stylophora* receptors. Within this cnidarian‐specific radiation, there are seven clades (clades VII‐XIII), with representatives from *Nematostella* and *Aurelia* in five, from *Stylophora* in four, and from *Hydra* in two clades, indicating a similar and partially overlapping diversification of inhibitory receptors in Anthozoa (*Nematostella* and *Stylophora*) and Medusozoa (*Hydra* and *Aurelia*).

Of the six further clades containing cnidarian pLGICs, five are cnidarian‐specific (clades II‐VI; Figure 2). While cnidarian‐specific clade V appears to be basal to the bilaterian GABA_A_ receptors, clade VI, containing three *Nematostella* receptors (NV2.24331, NV2.3907, and NV2.13369), is placed in a sister clade to bilaterian GABA_A_Rs. Notably, two other *Nematostella* receptors (Nv2.17304 and Nv2.15844) robustly cluster together with one *Hydra* and five *Platynereis* receptors in clade I at the base of the GABA_A_R/GlyR superfamily with good support from maximum likelihood analysis (bootstrap 100%; Figure 2).

In conclusion, our analysis suggests a complex evolution of inhibitory pLGICs. Notably, bilaterian GABA_A_ receptors form a monophyletic clade within the superfamily, derived from a common bilaterian‐cnidarian ancestor at the base of clade VI. In addition, our analysis reveals a massive cnidarian expansion of pLGICs that contains seven clades. Furthermore, cnidarian pLGICs in clades III and IV seem to be sister to a *C. elegans*‐specific clade that contains receptors activated by different ligands, but not by GABA. Finally, the robust position of a small cluster of two *Nematostella*, one *Hydra*, and five *Platynereis* receptors in clade I at the base of cnidarian/bilaterian GABA_A_R/GlyR superfamily suggests that these have split early from the rest of the superfamily. Because of this complex phylogenetic history, automated gene annotations based only on sequence similarity should be taken with caution and ligand specificity cannot be easily predicted from the phylogenetic analysis alone.

### 2.2 Some Putative GABA_A_Rs in *Nematostella* form Homopentamers

We next assessed the expression profile of all LGICs related to GABA_A_Rs, using our single‐cell dataset [18]. We found that most of the receptors are expressed either in POU4‐positive (N2) neurons, cnidocytes or tentacle retractor muscles (Figure S2). As the feeding response involves the use of cnidocytes, neurons and retractor muscles in the tentacles, we therefore focused on subunits expressed in these cells of the tentacles (see below). We cloned seven subunits from *N. vectensis*, referred to here as nvpLGIC‐1 –nvpLGIC‐7 (red or bold in Figure 2), belonging to five of the seven gene clades of the cnidarian‐specific radiation. nvpLGIC‐1 – nvpLGIC‐7 are 405–490 amino acids in length (Figure S3). Sequence alignment with the human GABA_A_ α1 and β3 subunits revealed that they contain the hallmarks of Cys‐loop receptors: an N‐terminal ligand‐binding extracellular domain (ECD), four transmembrane helices (M1‐M4), an intracellular loop between M3 and M4, an extracellular C‐terminal domain, and a disulfide bond in a loop at the interface between the ECD and the transmembrane domain, which identifies them as Cys‐loop receptors (Figure 1; Figure S3). Human α1 GABA_A_ subunits form homopentamers when a residue in either M1 or M3 is mutated to Trp [31], and an intramembrane aromatic network determines the assembly of pLGICs [32]. nvpLGIC‐1 – nvpLGIC‐7 contain a Trp residue at the critical position in M1 (Figure S3), suggesting that they might form homopentamers.

**FIGURE 3:**
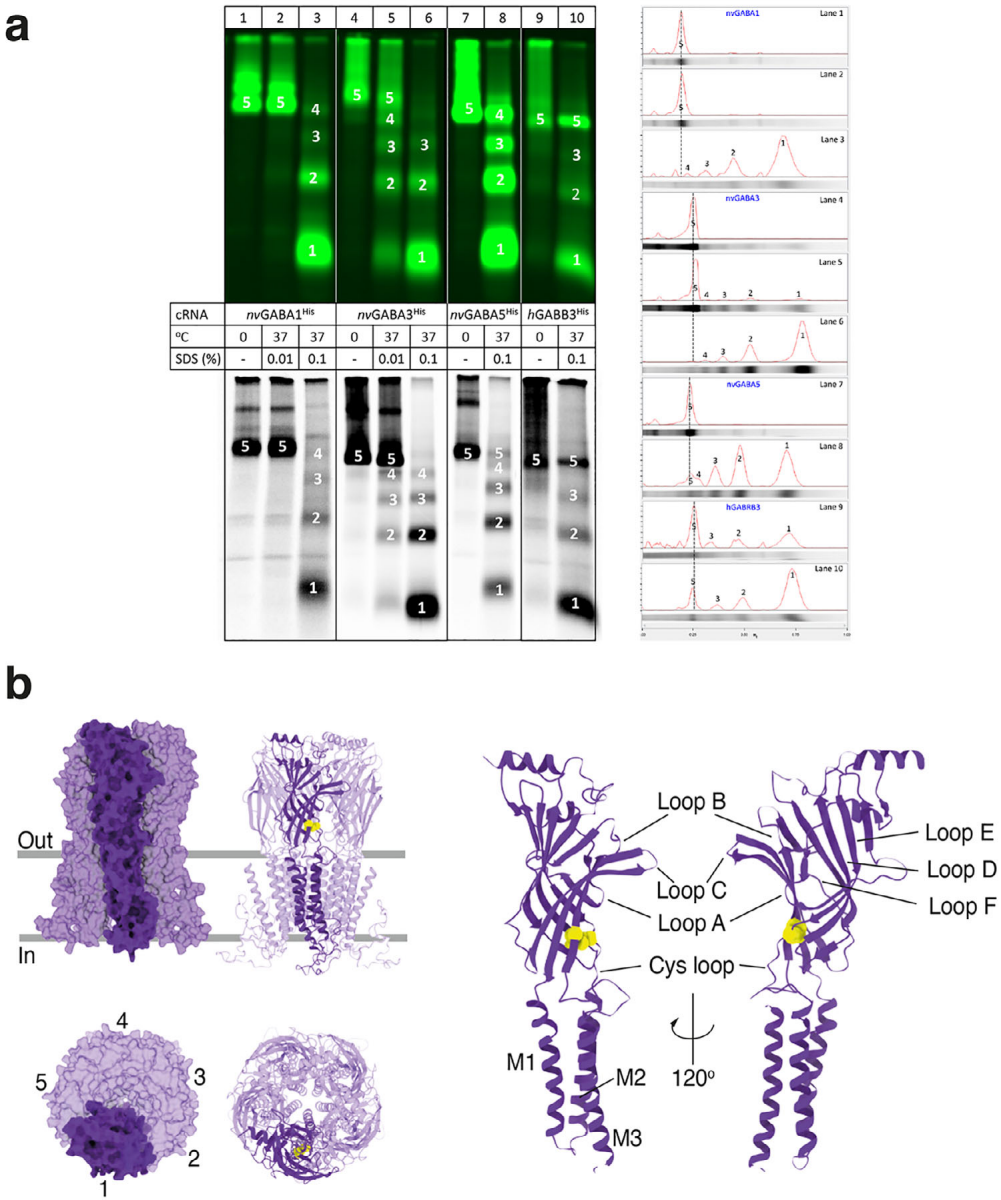
*Nematostella* pLGICs form homomeric Cys‐loop receptors. a) Visualization of the oligomeric state of nvpLGICs versus pentameric human GABA_A_ β3. Top left, IR800 fluorescence detecting the surface‐expressed pool of receptors. Bottom left, ^35^S‐labelled proteins detecting the total pool. Right, IR800 fluorescence was quantified along each lane, which is also shown horizontally in grey. Peaks are numbered according to the protein bands on the left. The close similarity of the band patterns to the similarly sized human β3 receptor identifies the nvpLGICs as homopentamers. b) Homology model of nvpLGIC‐2 based on PDB entry 4COF. Left, overview of the homopentameric complex. A single subunit is highlighted. Top, side view; bottom, top view. Right, domain structure of a single subunit. S─S bonds are shown as yellow spheres. Loops contributing to the ligand binding site are indicated. Presentation based on Allard et al. [34].

To directly test the assembly and formation of homopentamers, we tagged three representative pLGICs (pLGIC‐1, pLGIC‐3, and pLGIC‐5) at their extracellular C‐termini with a hexahistidine‐tag and analyzed their plasma membrane expression and oligomeric assembly state using denaturing SDS‐PAGE and non‐denaturing blue native PAGE after expression in *Xenopus laevis* oocytes. As a positive control, we used the human GABA_A_ β3 subunit, hGABRB3, which assembles as a functional homopentamer, the structure of which has been solved [33]. We purified the histidine‐tagged versions of these receptors under non‐denaturing conditions using Ni‐NTA chromatography. The total pool of GABA subunits was metabolically labelled with [^35^S]methionine, whereas the plasma membrane‐bound pool was labelled at the cell surface of intact oocytes by the lysine‐reactive infrared dye IR800‐NHS ester. The purified nvGABA receptors migrated both in the plasma membrane form (Figure 3a) and in the whole cell form as distinct high‐mass proteins that dissociated into lower‐order intermediates when exposed to the denaturing detergent SDS for 1 h at 37°C (Figure 3a). The maximum number of five bands generated indicates that all three nvGABA receptors assemble as homopentamers, identical to hGABRB3. The similarity of the band patterns reflects the similar subunit masses of the four proteins. Using the crystal structure of the human β3 homopentamer [33] as a template, we generated a homology model of homomeric nvpLGIC‐2, revealing a canonical pLGIC structure with a Cys‐loop and several loops that form the ligand‐binding site (Figure 3b).

### 2.3 Functional Expression Revealed Three Glutamate‐Gated Cl^−^‐Channels (nvGluCls) Among Putative GABA_A_ Receptors

**FIGURE 4:**
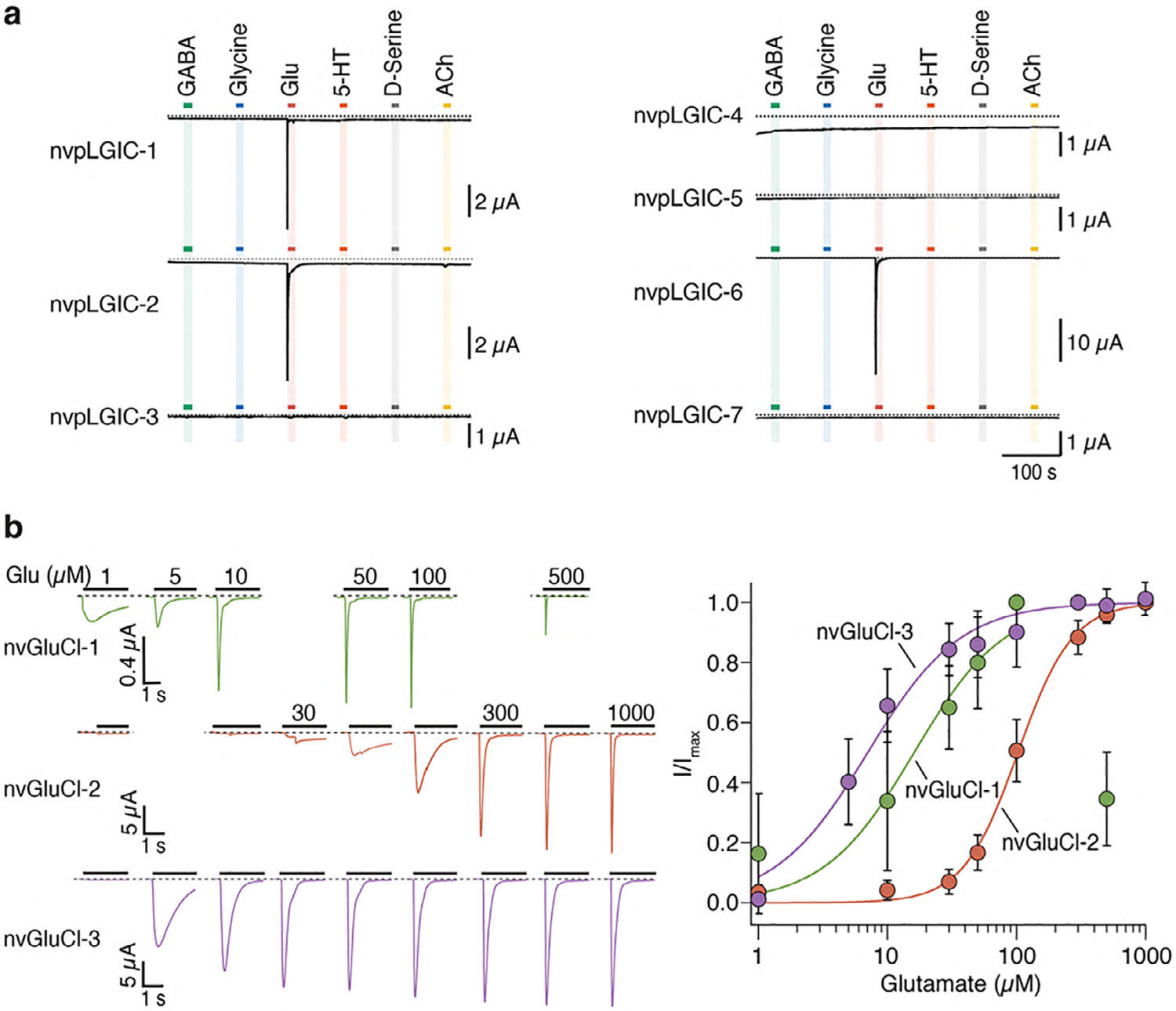
Three *Nematostella* pLGICs are activated by micromolar concentrations of glutamate. a) representative current traces from oocytes expressing nvpLGIC‐1 to nvpLGIC‐7. The indicated ligands were applied at a concentration of 1 mm, except for glutamate, which was applied at 100 µm. Dashed lines represent the 0 current level. b) Left, representative current traces showing activation of nvGluCl‐1 (nvpLGIC‐1), nvGluCl‐2 (nvpLGIC‐2), and nvGluCl‐3 (nvpLGIC‐6) upon application of the indicated concentrations of glutamate (black bars). Right, concentration‐response curves for nvGluCl‐1 (green), nvGluCl‐2 (orange), and nvGluCl‐3 (violet). Data represent mean normalized currents ± SD from 6–17 cells. Solid lines indicate fits to the Hill function.

We expressed nvpLGIC‐1 – nvpLGIC‐7 individually in *Xenopus* oocytes and functionally characterized them using two‐electrode voltage clamp. Surprisingly, neither GABA nor glycine (1 µm–1 mm) evoked currents in nvpLGIC‐expressing oocytes. We then considered glutamate, the precursor of GABA, as a putative small‐molecule agonist. Interestingly, glutamate evoked large currents in oocytes expressing nvpLGIC‐1, nvpLGIC‐2, and nvpLGIC‐6, but not in oocytes expressing nvpLGIC‐3, nvpLGIC‐4, nvpLGIC‐5, or nvpLGIC‐7 (Figure 4a). Similarly, serotonin (5‐HT), D‐serine, and acetylcholine did not elicit currents with nvpLGIC‐1 – nvpLGIC7 (Figure 4a), nor did aspartate, glutamine, histamine, or β‐alanine, not even with a mix of nvpLGIC‐1 – nvpLGIC‐7 (we did not test nvpLGIC‐7 individually with the latter agonists). Therefore, we renamed the three receptors responding to glutamate nvGluCl‐1 (nvpLGIC‐1), nvGluCl‐2 (nvpLGIC‐2), and nvGluCl‐3 (nvpLGIC‐6), respectively. These three receptors form a monophyletic clade within the cnidarian GABA_A_ family (Figure 2).

We continued to focus on the glutamate receptors. Application of increasing glutamate concentrations revealed that concentrations needed for half‐maximal activation of the three receptors varied more than 10‐fold, ranging from 7 to 100 µm (EC_50_ = 16 µm for GluCl‐1, EC_50_ = 102 µm for GluCl‐2, and EC_50_ = 7 µm for GluCl‐3, respectively; Figure 4b). Glutamate‐evoked currents desensitized completely. With increasing glutamate concentrations, desensitization became more rapid until it reached a plateau at saturating glutamate concentrations. We determined the time constant of desensitization, τ_des_, at 500 µm glutamate with a fit to a single exponential function, revealing τ_des_ = 26 ± 3 ms for nvGluCl‐1, 111 ± 47 ms for nvGluCl‐2, and 161 ± 93 ms for nvGluCl‐3, respectively (Figure S4a). It is likely that these time constants are limited by the comparatively slow solution exchange (10‐90% solution exchange ∼300 ms [35]) of our setup for *Xenopus* oocytes. However, these values are similar to those of mammalian GABA_A_Rs, which typically desensitize with a fast component on the order of 2–50 ms and a slow component on the order of 50 ms to several seconds, depending on the subunit stoichiometry [36, 37, 38]. For nvGluCl‐2 and nvGluCl‐3, we also determined recovery from desensitization, which had a similar τ_recovery_ of 31 sec and 38 sec, respectively (Figure S4b).

**FIGURE 5:**
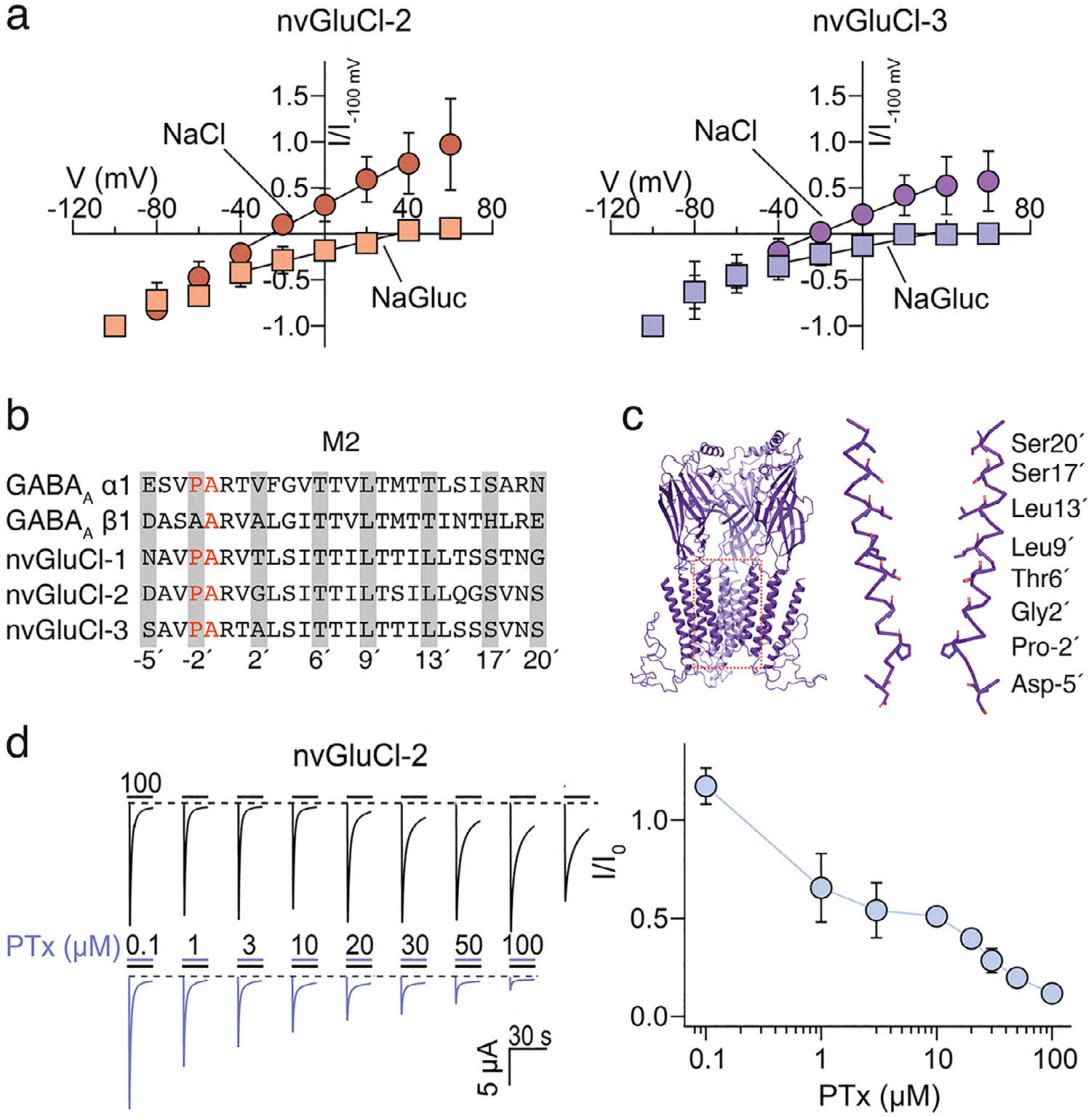
nvGluCls are chloride channels. (a) I‐V relationships for nvGluCl‐2 (left) and nvGluCl‐3 (right) in control bath solution (circles) and in a bath solution, in which NaCl was replaced by NaGluconate (NaGluc; squares). nvGluCl‐2 was activated with 500 µM glutamate, and nvGluCl‐3 (right panel) with 50 µm glutamate. Data are plotted as mean ± SD for 3–15 cells. Data points were fitted with a linear function (black lines) to obtain the reversal potential (*E*_rev_). For nvGluCl‐2, *E*_rev_ was −25 mV in control solution and 34 mv with NaGluconate, and for nvGluCl‐6, *E*_rev_ was −21 mV in control solution and 29 mV with NaGluconate. (b) Sequence alignment of M2 sequences of human GABA_A_ α1, human GABA_A_ β3, and nvGluCl‐1 – nvGluCl‐3. The cytoplasmic side is to the left, and the extracellular side is to the right. Residues that line the ion pore are shown on a grey background and numbered according to the convention for Cys loop receptors. They form rings of identical residues in five‐fold symmetry of functional homomeric receptors. The ‐1´Ala and ‐2´ Pro residues are shown in red. (c) Homology model of nvGluCl‐2. The front subunit has been removed for clarity, and the indicated region is shown on an expanded scale on the right. Only two M2 helices are shown. Amino acids facing the ion pore are shown as sticks. (d) Left, representative current traces for nvGluCl‐2 obtained upon application of 100 µM glutamate with and without the indicated concentrations of picrotoxin (PTx). Right, concentration‐response curve. Data represent the mean ± SD of 6–16 cells.

To assess the ion selectivity of nvGluCl‐2 and nvGluCl‐3, we determined the reversal potential E_rev_ with 140 mm NaCl in the extracellular solution, revealing an E_rev_ of ∼−20 mV, close to the reversal potential of Cl^−^ in oocytes under these ionic conditions. When Cl^−^ in the extracellular solution was replaced with the large impermeant anion gluconate, E_rev_ shifted to ∼+30 mV (Figure 5a), confirming that Cl^−^ was the main permeant ion. The ion pore of Cys‐loop receptors is lined by residues of the five M2 α helices. Figure 5b shows an alignment of the M2 sequences of nvGluCl‐1, nvGluCl‐2, and nvGluCl‐3, together with human α1 and β3 GABA_A_ subunits. The open ion pore of pLGICs has its strongest constriction at the ‐2´ residue [33, 39]. Determinants of anion selectivity are ‐1´Ala and ‐2´ Pro residues, whereas cation channels have a ‐1´ Glu residue [39], and often lack the ‐2´ residue. In nvGluCl‐1 – nvGluCl‐3, ‐1´Ala and ‐2´ Pro are conserved, consistent with the finding that they are glutamate‐gated Cl^−^ channels. In addition, the homology model of nvGluCl‐2 confirmed the constriction at Pro‐2´ and the position of residues lining the ion pore (Figure 5c).

To further test the conservation of the nvGluCl ion pore with the ion pores of GABA_A_Rs, we assessed the inhibition of nvGluCl‐2 by picrotoxin (PTx), an open‐channel blocker of GABA_A_Rs. PTx binds to the cytosolic base of the pore, close to M2 residues ‐2´and 2´ in *Caenorhabditis* GluClα [39] or between the M2 2´ and 9´ rings in the heteromeric human α1β3γ2 GABA_A_R [40]. In the human receptor, the hydrophobic isoprenyl moiety of PTx is surrounded by the 9´ Leu ring, and the oxygen atoms of PTx form putative hydrogen bonds with the 6´ Thr ring. The conservation of the 6´ Thr and 9´ Leu rings in nvGluCls (Figure 5b,c) strongly suggests that they could also bind PTx. PTx indeed inhibited nvGluCl‐2 with an apparent IC_50_ of ∼10 µM (Figure 5d), similar to canonical GABA_A_Rs, illustrating the conserved overall architecture of the nvGluCl ion pore with that of GABA_A_Rs.

### 2.4 Amino Acid Residues that Determine the Ligand Sensitivity of nvGluCls

**FIGURE 6:**
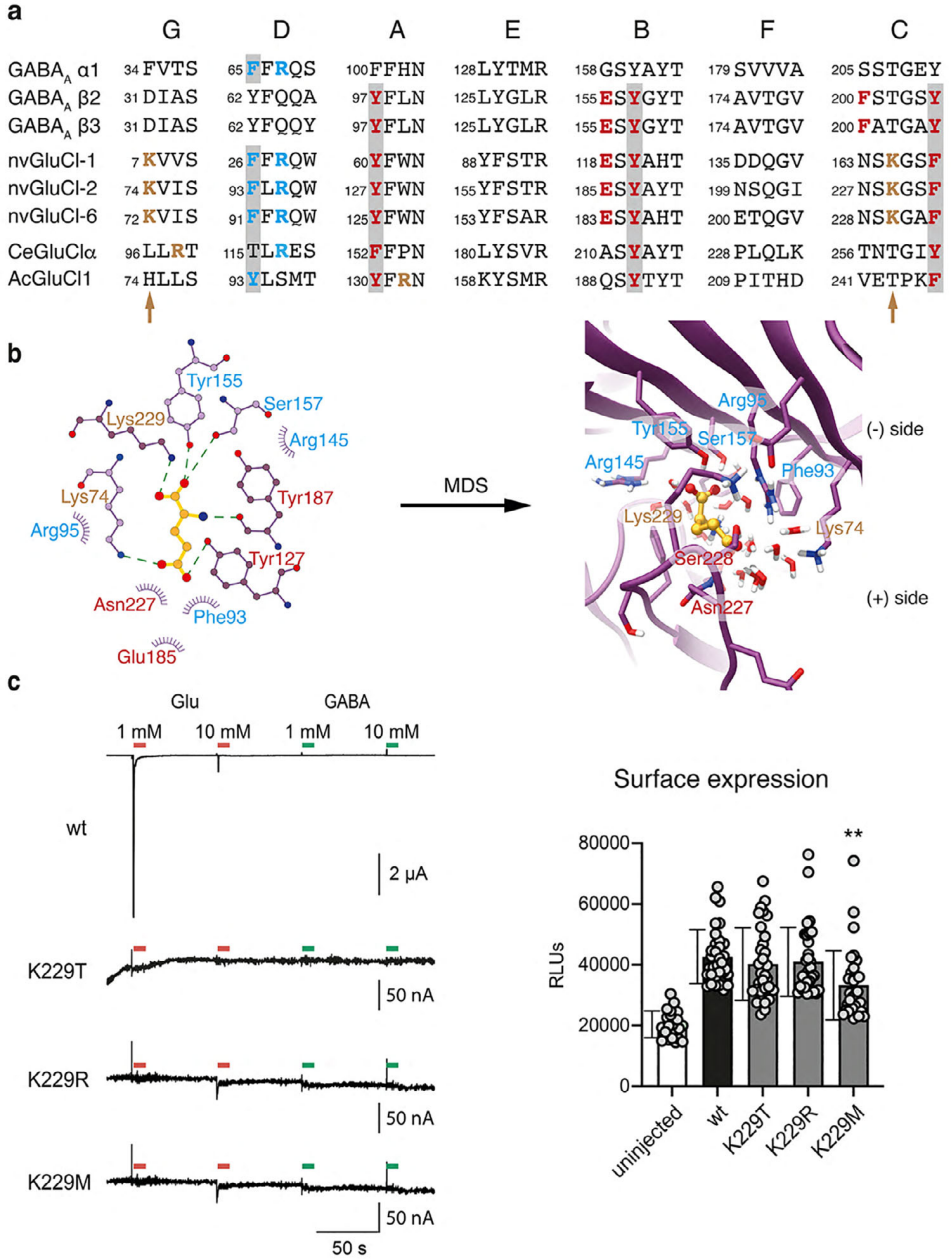
A lysine residue in loop C is essential for glutamate binding. (a) Sequence alignments of the loops forming ligand‐binding pockets of human α1, β2, and β3 subunits, of nvGluCls and GluCls from *Caenorhabditis elegans* and *Aplysia californica*. Crucial residues of the principal β subunits are shown in red, and those of the complementary α subunits are shown in blue; residues of the aromatic box are on grey background. The critical Lys residues in loops C and G of nvGluCls are shown in brown (arrows). (b) Left, result from docking Glu onto nvGluCl‐2. LIGPLOT schematic of Glu interactions. Electrostatic interactions are shown as dashes and hydrophobic interactions as eyelashes.  Residues of the principal and complementary subunits are shown in red and blue, respectively. K74 and K229 are shown in brown; K74 is from the complementary subunit, and K229 is from the principal subunit. Right, binding mode of Glu (yellow, ball‐and‐stick representation) in the most representative structure from molecular dynamics simulation (MDS) of wild‐type nvGluCl‐2 replica 2. Residues within 0.5 nm of glutamate are shown in purple licorice representation. (c) Loss of function of nvGluCl‐2 with substitutions of K229. Left, representative current traces. Right, surface expression of His‐tagged nvGluCl‐2 wt. and mutants. Results are expressed as relative light units (RLUs) per oocyte. Bars represent the mean, and error bars, which are shown to the left of the bars, the SD. All conditions had higher RLUs than uninjected oocytes, which served as a control. Control, *n* = 24; K229T, *n* = 31; all others, *n* = 32. ^**^, P < 0.001 (compared to wt.; one‐way ANOVA).

We next addressed the question of which specific amino acids in the ligand‐binding pocket are responsible for glutamate versus GABA binding in nvGluRs. The ligand‐binding pocket of Cys‐loop receptors localizes at the interface of two neighboring subunits. One subunit contributes the principal or (+) side, and the other the complementary or (‐) side (Figure 1). The binding pocket is mainly formed by non‐contiguous loops A‐C on the (+) side and two β‐strands and one loop (“loops” D‐F) on the (‐) side (Figures 3b and 6a) [41]. Human α1β2γ2 and α1β3γ2 GABA_A_Rs contain two α subunits and two β subunits, and the GABA‐binding pockets are located at the two β‐α interfaces, where the β subunits contribute the (+) sides (Figure 1). GABA is surrounded by an “aromatic box” formed by Y97, Y157, F200, and Y205 of the β subunit, and F65 of the α subunit. While the GABA amino group is engaged in a cation‐π interaction with β‐Y205 of loop C and is further stabilized by hydrogen bonds with β‐E155 of loop B, the γ‐carboxylate of GABA forms a salt bridge with α‐R67 of loop D [40, 42]. Overall, key amino acids of the ligand‐binding pocket are surprisingly well conserved in the three nvGluCls (Figure 6a). In particular, the Arg residue of loop D and the aromatic box are completely conserved, except for the residue corresponding to β‐F200 of loop C, which is replaced by a polar Asn residue. Moreover, nvGluCls have a Lys residue in loop C (K229 in nvGluCl‐2; Figure 6a), close to the site where the basic GABA amino group binds in human GABA_A_Rs. This lysine should increase the positive electrostatic potential of the binding pocket and might help to stabilize the additional α‐carboxylate group of the glutamate ligand. The homology model of nvGluCl‐2 confirmed that loops A‐F could form a classical neurotransmitter binding site (Figure 3b). To obtain a better impression of the potential Glu‐binding site in nvGluCls, we docked Glu to the nvGluCl‐2 homology model (Figure S5a). Strikingly, the best binding pose indicated a salt bridge between the α‐carboxylate of Glu and K229 of loop C. In this model, R67 of loop D forms a salt bridge with the α‐carboxylate of Glu, instead of the γ‐carboxylate of the ligand as in GABA_A_Rs. In the nvGluCl‐2 model, the γ‐carboxylate of Glu rather forms a salt bridge with K74 of a different region (“loop G”) from the (‐) subunit (Figure 6b, left). Interestingly, in GluClα of *C. elegans*, an Arg residue of loop G (Figure 6a) interacts with the α‐carboxylates of glutamate [39]. Thus, it appears that the glutamate ligand is anchored in the ligand‐binding pocket of nvGluCl‐2 via electrostatic interactions between its two carboxylates and two Lys residues of the receptor (Figure 6b, left). To test the contribution of K229 to ligand binding in nvGluCl‐2, we mutated it to threonine, the residue found in human β subunits, to arginine, and to methionine, two more conservative substitutions, and found that these mutations almost completely abolished the response to high concentrations of glutamate (10 mm; Figure 6c), but did not reduce expression of mutant channels at the cell surface, except for K229M (Figure 5c), the expression of which was still above control cells, confirming that K229 makes an essential contribution to glutamate binding. Mutant channels were still not activated by GABA (Figure 6c), indicating that other residues are also important for the ligand switch. Importantly, a Lys residue is present in loops C and G of nvGluCl‐1 – nvGluCl‐3, but not of nvpLGIC‐3, nvpLGIC‐4, nvpLGIC‐5, or nvpLGIC‐7 (Figure S3), further suggesting that these residues are important for glutamate binding in nvGluCls and that nvpLGICs lacking these Lys residues have another ligand.

We also evaluated the stability of the model obtained from molecular docking by performing triplicate atomistic molecular dynamics simulations up to 200 ns in explicit solvent for both the K229T mutant and the wild‐type nvGluCl‐2 channel. In the K229T mutant, glutamate dissociated from the binding pocket after 149, 72, and 86 ns, respectively (Figure S5b). In the wild‐type channel, ligand dissociation was observed in two of the three replicas at 111 ns and 124 ns, while in the third trajectory, glutamate remained bound throughout the simulation in an equilibrated and optimized binding mode despite the flexibility of surrounding residues (Figure S5b,c). The most representative structure from this simulation shows that the ligand preserved the orientation and position predicted by docking, but that some specific interactions changed. Notably, K229 did not maintain a direct interaction with the ligand, which instead became mediated by a hydrogen‐bond network involving S228. At the same time, K229 established a new salt bridge with E72 of the (‐) side (Figure 6b, right), suggesting a second alternative role for K229 in favoring glutamate binding through pocket stabilization rather than direct electrostatic coordination. Other key contacts identified in the docking pose were preserved for Y155 and S159, while the interaction involving K74 became water‐mediated, and two additional hydrogen bonds formed with N227 and S228 (Figure 6b, right).

### 2.5 Cnidarian‐Specific pLGICs are Expressed in Cnidarian‐Specific Cell Types and Neurons

**FIGURE 7:**
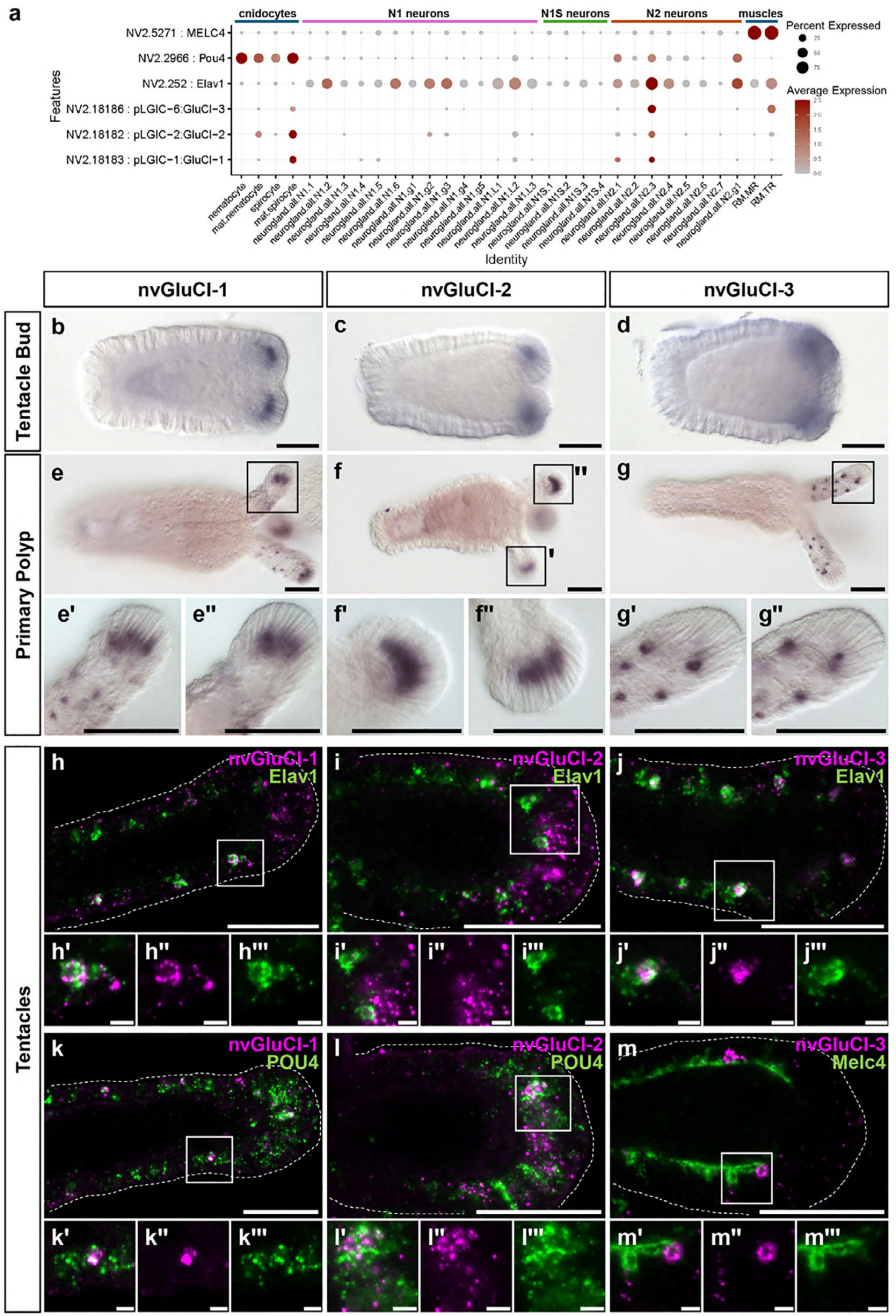
pLGIC expression is predominately in cells of the tentacle and individual class N2 neurons. (a) Dotplot of expression profiles within cnidocytes, neurons, secretory cells, and fast muscle of the three glutamate‐responsive receptors investigated in this study. Expression in early cell states is not shown. See Figure S2 for expression profiles of all genes across the entire dataset. (b–g) In situ hybridization showed expression within cells of the tentacles from the tentacle bud to the primary polyp stage (indicated on the left). All images show lateral views with the aboral pole to the left. Scale bars, 50 µm. (h–m) Double fluorescent in situ hybridization for GluCl (magenta) and cell type‐specific markers (green) confirmed expression of nvGluCl‐1 and nvGluCl‐3 in elav1‐positive neurons (h, j), and nvGluCl‐1 and nvGluCl‐2 in pou4‐positive cells, (k, l), and nvGluCl‐3 in cells closely associated with the melc4‐positive tentacle retractor muscle (m). Images are confocal sections showing lateral‐view tentacles of primary polyps with tentacle tips to the right. Scale bars, 50 µm overview, 5 µm enlarged section.

We assayed the expression profiles of the nvpLGICs in the available single‐cell atlas [18] and found that 36 of them are specifically expressed at detectable levels across various cell states (Figure S2). Expression was predominantly found within mature cnidocyte profiles and related N2 class neurons, defined by the expression of Brn3/POU4 transcription factor family gene [18]. nvGluCls were detected within cell types specific to the tentacles of the polyp, including both spirocytes, an anthozoan‐specific cnidocyte cell type [43], and tentacle retractor muscles (Figure 7a). nvGluCl‐3 was also detected in one specific Pou4‐positive neural subtype (N2 neuron 3), whereas pLGIC‐5 is expressed in both mature spirocytes and the FoxQ2d‐positive N1S neuron 4 (Figure S2) [18, 44]. Notably, several tetrameric ionotropic glutamate receptors (iGluRs), considered to be cation‐selective (excitatory) receptors, are co‐expressed with one or more of the nvGluCls in a few cell types (e.g., mature spirocytes and N2.3 neurons; Figure S6).

To validate the predicted gene expression from single‐cell RNA‐seq analyses, we generated in situ hybridization probes for the gene models of the three nvGluCls. We found expression of all three genes from the late planula stage, which accumulate in the tentacle bud stage in the growing tentacles during metamorphosis into the primary polyps (Figure 7b–d). Once the tentacles have elongated, nvGluCl‐1 and nvGluCl‐3 are expressed in individual cells at the base of the ectoderm (Figure 7g), which is indicative of expression in tentacle neurons and tentacle retractor muscle cells. In addition, nvGluCl‐1 and nvGluCl‐2 are expressed in ectodermal cells at the tentacle tip (Figure 7e,f). This spatial distribution is characteristic of spirocytes, a type of cnidocyte that is restricted to the tentacles.

To confirm the cell types in which the three receptors are expressed, we performed double fluorescence in situ hybridization (Figure 7h,i). *Elav1* has been previously reported to be expressed in a subpopulation of neurons [15], whereas *POU4* is expressed in all cnidocytes and class N2 neurons [17, 45]. Double FISH showed that nvGluCl‐1 and nvGluCl‐3 are expressed in a subpopulation of the *Elav1*‐positive neurons (Figure 7h,j). Furthermore, nvGluCl‐1 and nvGluCl‐2 are expressed in a fraction of the *POU4*‐positive cells (Figure 7k,l). The co‐expression in *POU‐4* positive, *elav1* negative cells at the tip of the tentacle is suggestive of nvGluCl‐1 and nvGluCl‐2 expression in spirocytes. We also investigated the expression of nvGluCl‐3 in the tentacle retractor muscle by double fluorescent in situ hybridization with *melc4* as a marker of fast‐retracting muscle [26] (Figure 7m). nvGluCl‐3 is expressed in cells closely associated with tentacle retractor muscle cells, presumably neurons innervating the tentacle retractor muscles. Occasional co‐expression with *melc4* cannot be ruled out because of its close co‐localization (Figure 7m). Together, these observations suggest that the three tested nvGluCl‐responsive receptors may be involved in the coordination of tentacle movement in response to prey.

## 3 Discussion

### 3.1 The Evolution of Inhibitory Signaling

Neuronal LGICs are fundamental for complex behaviors and rapid sensory processing. However, the evolution of neurotransmitter signaling through LGICs in animals remains an unsolved mystery. Ionotropic glutamate receptors (iGluRs) have an ancient evolutionary origin, as they are also present in plants [46] and likely evolved from prokaryotic ion channels [47]. The origin and ancient ligands of pLGICs are less clear. Some procaryotes have pLGICs [48], and the procaryotic pLGIC ELIC is activated by GABA with low affinity (EC_50_ = 21 mm) [49]. However, eukaryotic pLGICs probably have a monophyletic origin [50], and their ancient ligand is unknown. Moreover, sequence analysis suggests that the split into cation‐ and anion‐selective pLGICs preceded the evolution of animals [51] and further implies gene loss in sponges and ctenophores. The occurrence of pLGICs in the sponge *C. candelabrum* could be an exception to this loss, or alternatively obtained secondarily by lateral gene transfer from another lineage. We cannot currently distinguish between these two alternatives.

Cnidarians contain divergent copies of genes coding for iGluRs and of genes with homology to AChRs, GABA_A_Rs, or GlyRs [13]. Our results reveal a surprising complexity of the genes with homology to GABA_A_Rs/GlyRs in cnidarians. Of the 39 *Nematostella* nvpLGICs that belong to the inhibitory pLGIC branch, two are forming a basally branching cluster with one *Hydra* and 5 *Platynereis* pLGICs. In our phylogenetic analysis, 26 *Nematostella*, 10 *Hydra*, 15 *Aurelia*, and 11 *Stylophora* inhibitory pLGICs form a large monophyletic cnidarian expansion that evolved independently from other clades containing bilaterian receptors. These findings have important implications for the evolution of inhibitory LGICs.

First, it remains unclear whether the ancestor of the inhibitory pLGIC family was gated by GABA. Bilaterian GABA_A_Rs form a monophyletic clade, probably derived from a common bilaterian‐cnidarian ancestor at the base of clade VI (Figure 2). Our phylogenetic analyses put 5 *Nematostella* receptors on sister clades to these bilaterian GABA_A_Rs with a fairly high support (83 and 97%, respectively). The specialization of vertebrate subunits, for example, into α and β subunits, likely occurred after this split. Whether the ancestor of this bilaterian‐cnidarian clade was gated by GABA remains an open question that needs to be addressed in the future by functional characterization of clade V and VI nvpLGICs. The ligands of ancestral channels at deeper nodes within the inhibitory pLGIC superfamily remain also uncertain.

GABA is an important inhibitory neurotransmitter in both vertebrates and invertebrates. While prokaryotic cells and plants can also synthesize GABA, where it serves as a metabolic product, the important question is when GABA was first used for neuronal signaling in animal nervous systems. *N. vectensis* possesses genes with homology to the key enzyme for GABA synthesis, glutamate decarboxylase (GAD), with a relatively low identity to the vertebrate enzymes [13, 51]. GABA immunoreactivity has been found in sensory and sub‐epithelial neurons of *N. vectensis* [52]. *N. vectensis* also has homologs of proteins required for GABA import into synaptic vesicles, presynaptic reuptake, and metabotropic GABA_B_ receptors (GABA_B_Rs), which contain conserved GABA‐binding sites and presumably mediate the effects of the GABA_B_‐specific agonist baclofen on neurogenesis [53]. Thus, metabotropic GABA_B_Rs likely mediate GABA signaling in *N. vectensis*. Homology‐based prediction of GABA_A_Rs in the *N. vectensis* genome led to the assumption that *N. vectensis* uses GABA also for fast ionotropic signaling. In TEVC experiments, of seven expressed LGICs, none was activated by GABA or glycine, but three were shown to be chloride channels activated by glutamate. Since all three are expressed in spirocytes, the tentacle retractor muscle or neurons in the tentacle, we speculate that the receptors might be involved in the control of the feeding response. Taken together, the combination of phylogenetic and functional analyses used in our study suggests that the radiation of pLGICs preceded the acquisition of ligand specificity, which evolved independently within the various clades and animal lineages. In summary, the answer to the question of the ancient ligand of channels of the GABA_A_R/GlyR superfamily remains open and awaits deorphanization of more cnidarian receptors within clade VI, sister to bilaterian GABA_A_Rs, and clade I, basal to all other inhibitory pLGICs.

### 3.2 The Diversity of Inhibitory LGICs in *Nematostella*

The second implication of our findings is that inhibitory pLGICs diversified extensively in cnidarians. In the phylogenetic analysis, it appears that all subunits of the cnidarian‐specific clade evolved from a single precursor. As we have shown here, three of these subunits are activated by Glu. We found that in the three nvpLGICs (NV2.181184, NV2.18185, and NV2.19245; Figure 2), which belong together with the nvGluCls to clade XIII, the two critical Lys residues (K74 and K228 in nvGluCl‐2) are conserved, strongly suggesting that these receptors are also activated by Glu. However, in most nvpLGIC subunits, these two lysine residues are absent, strongly suggesting that they have another ligand, as we demonstrated for pLGIC‐3, pLGIC‐4, pLGIC‐5, and pLGIC‐7. For these four subunits, this ligand is unlikely to be GABA or glycine. The sequence variations in the ligand‐binding pockets of the cnidarian pLGICs of the GABA_A_ type suggest the existence of a variety of ligands that are yet to be identified.

We found that nvGluCl‐1, nvGluCl‐2, and nvGluCl‐3 are anion channels like human GABA_A_Rs. This finding is in agreement with the presence of ‐1´Ala and ‐2´ Pro residues in M2 [39]. Interestingly, while also nvpLGIC‐3, nvpLGIC‐4, and nvpLGIC‐5 possess ‐1´Ala and ‐2´ Pro in M2, nvpLGIC‐7 and several other nvpLGICs of the same *Nematostella*‐specific clade VIII have an Asp residue at position ‐1´ (Figure S3), which forms a ring of negative charges within the ion pore, strongly suggesting that nvpLGICs of this clade are cation channels. Together, these findings suggest a scenario in which, within the cnidarian radiation, ligand specificity and ion selectivity of pLGICs rapidly diversified starting from a single precursor. We speculate that this specialization of ion channel receptors was instrumental in the evolution of complex behaviors and sensory processing by the cnidarian nervous system [54]. In particular, the diversification of anion channels may have allowed the refinement of inhibitory systems.

Interestingly, lophotrochozoan invertebrates also have inhibitory pLGICs gated by diverse ligands, such as glutamate (GluCls) [1, 2, 3], serotonin (MOD‐1) [4] and other biogenic amines [5], acetylcholine (ACC, LGC‐46) [6], or histamine [7]. Thus, it appears that the relatively uniform ionotropic inhibitory signaling with just two main ligands (GABA and glycine), which is characteristic of vertebrate nervous systems, is the exception rather than the rule in the animal kingdom. We note, however, that also in vertebrates, glutamate allosterically potentiates GlyR currents and modulates GABA_A_Rs [55, 56] and that histamine modulates GABA_A_Rs [57, 58], showing that these receptors bind more than just GABA and glycine.

We assume that anion‐selective cnidarian pLGICs are inhibitory, but note that this may not always be the case. Depending on the Cl^−^ equilibrium potential E_Cl_ and the cell membrane potential E_M_, opening of a Cl^−^ channel will hyperpolarize or depolarize a cell. If E_Cl_ is close to E_M_, the increased conductance of an open Cl^−^ channel shunts excitatory currents, thereby inhibiting a neuron even if E_Cl_ is slightly more positive than E_M_. However, if E_Cl_ is much more positive than E_M_, a Cl^−^ current can also be excitatory. Future studies need to confirm whether nvpLGICs are inhibitory or, in some cases, excitatory.

### 3.3 Convergent Evolution of Glutamate‐Gated Ion Channels

It is generally believed that glutamate was used as a transmitter for fast ionotropic signaling already at the base of the animal kingdom [12, 59]. As a proteinogenic amino acid, it is ubiquitous; there are homologs of vesicular transporters and reuptake transporters, and a large variety of predicted iGluRs in the *N. vectensis* genome [13, 51]. While formal proof that these receptors bind to and are activated by glutamate is missing, our study establishes glutamate‐gated ion channels in the cnidarian pLGIC superfamily. Of note, K74 and K229, which are essential for Glu binding in nvGluCl‐2 (Figure 5), are absent from GluCls in *C. elegans* (Figure 5a). These GluCls use another Arg residue of loop G to stabilize the α‐carboxylates of glutamate [39]. Furthermore, yet another Arg residue in loop A is important for glutamate binding in molluscan GluCls (Figure 5a), indicating that Glu‐binding pockets have evolved several times independently in pLGICs [60]. This is also consistent with these receptors belonging to different clades (Figure 2). Interestingly, while GABA binds to the β+/α− interface of mammalian GABA_A_Rs (Figure 1), it has recently been found that glutamate binds to the α+/β− interface to modulate the response of mammalian GABA_A_Rs to GABA [56]. Thus, it appears that there is an extensive crosstalk of glutamate with inhibitory GABA_A_Rs across the phylogenetic tree. Some GABA_A_Rs are modulated by Glu, while others (the GluCls) are directly activated by Glu.

### 3.4 Expression and Putative Functions of Glutamate‐Gated pLGICs in Nematostella

In situ hybridization confirmed that all three investigated nvGluCls are expressed in the tentacles – predominantly in cnidocytes, N2 neurons, or both, and to some extent also in tentacle retractor muscles. Single‐cell transcriptome analyses suggest expression of some nvGluCls in specific neuronal subpopulations, which may also be tentacle‐specific. The cnidocytes fall into several different types, including nematocytes and spirocytes; spirocytes are restricted to the tentacles. Nematocytes integrate multiple signals and receive synaptic input from spirocytes and sensory neurons [61]. Interestingly, while neither Glu nor GABA elicit currents in nematocytes, acetylcholine (ACh) elicits an inward Ca^2+^ current via nAChRs, which leads to the activation of a K^+^ channel and relieves inhibition of voltage‐gated Ca^2+^ channels (Ca_v_s), allowing the discharge of the nematocytes [61]. In addition, it has been found that the proton‐sensitive ion channel NeNaC2 can induce discharge of cnidocytes [62], illustrating that several transmitters act on cnidocytes. Therefore, we speculate that nvGluCl‐2 is expressed in spirocytes, where its activation by glutamate could hyperpolarize the neuron either to release Ca_v_s from inhibition, allowing discharge, or to inhibit discharge. The tentacle retractor muscle is the only ectodermal muscle in *Nematostella* and is one of the two fast‐contracting muscles [26]. Similar to the discharge of nematocytes, the contraction of the tentacle retractor muscles is regulated by ACh [63], and at least some nAChRs are also expressed in tentacles, similar to the nvGluRs in this study. Thus, nvGluCl‐2 and, in particular, nvGluCl‐3 are likely involved in the regulation of tentacle retractor muscle contraction. It is still unclear which cells secrete glutamate to bind to the nvGluCls characterized here in the tentacles. Interestingly, in *Hydra*, it has recently been postulated that bacteria located in the glycocalyx of the ectoderm, might be the source of glutamate to activate specific neuronal populations of the tentacle involved in the feeding response [64]. Thus, an exogenous source can also not be ruled out in *Nematostella*.

## 4 Conclusion

In summary, our study uncovers a complex phylogeny of inhibitory pLGICs and lays the basis for the functional characterization of ion channel receptor diversity in Cnidaria. Moreover, it emphasizes that the overall homology of ion channel receptors does not allow to predict their ligand specificity, which requires detailed structural and functional investigations.

## 5 Methods

### 5.1 Phylogenetic Analysis

Cnidarian pLGICs were retrieved from *N. vectensis* [29], *H. vulgaris* [30], *Aurelia coerulea* [65] and *Stylophora pistillata* (Uniprot) proteomes based on the InterproScan annotation as belonging to either the “Ligand‐gated ion‐channel” or the “Neurotransmitter‐gated ion‐channel” (IPR036734, IPR036719 and IPR038050) superfamilies. *Homo sapiens*, *Drosophila melanogaster*, *Caenorhabditis elegans, Platynereis dumerilii* [66] and *Branchiostoma floridae* pLGICs were retrieved from Swiss‐Prot or from the *Platynereis* genome resource (https://platynereis.com/resources/genome/). Datasets per species were deduplicated with CD‐hit v4.8.1 [67] using exhaustive search (g = 1) and 99% sequence similarity (c = 0.99). Deduplicated sequences were aligned using Mafft v7.526 [68] with the “localpair” algorithm and 1000 iterations (–maxiterate 1000 –localpair). The alignment was trimmed with Trimal v1.5.0 [69], using the “gappyout” algorithm (‐gappyout). The trimmed alignment was then used for tree construction using Iqtree2 v2.3.5 [70] with extended model selection (‐m MFP) and 10000 ultrafast bootstrap calculation (‐B 10000). Tree visualization was done using ggtree v3.12.0 [71].

All publicly available and annotated porifera and ctenophora genomes in NCBI were used for a comprehensive search of putative pLGICs using NCBI blast+ searches, InterProscan functional annotation and filtering as described above. For porifera, the genomes of *Oscarella* (GCF_947507565.1), *Sycon* (GCF_964019385.1), *Amphimedon* (GCF_000090795.2) and *Corticium* (GCF_963422355.1) were assessed, and for Ctenophora, the genomes of *Mnemiopsis* (GCA_048537945.1) and *Bolinopsis* (GCF_026151205.1).

### 5.2 Molecular Biology

Full‐length DNA sequences of GABA_A_ subunits were identified and cloned based on bulk and single cell transcriptomes of *N. vectensis* [17, 18, 26]. nvpLGIC‐1 – nvpLGIC‐4 were cloned in oocyte expression vector pRSSP6009 [72]. nvpLGIC‐5 and nvpLGIC‐7 DNA sequences were synthesized and cloned in the pCDNA3.1 (‐) vector (BioCat GmbH). They were then subcloned in the vector pRSSP6009 (nvpLGIC‐6) or pRSSP 6013 (nvpLGIC‐5). Site‐directed mutations were introduced in nvGluCl‐2 to create the mutants at position K229, using the Quick‐change mutagenesis method. nvpLGIC‐1 – nvpLGIC‐6 subunits were fused with His_6_‐tags at their C‐terminus using PCR. His_6_‐tags were incorporated just before the stop codon without changing any other amino acids. All final constructs were sequenced for confirmation. The plasmid containing full‐length cDNA for human GABRB3 was purchased from the Harvard Plasmid Repository (order number HsCD00043103; repository closed in January 2021) and subcloned into a Gateway‐compatible version of the pNKS2 vector [73] using the Gateway cloning system (Invitrogen, Karlsruhe, Germany). cRNA was synthesized using SP6 RNA polymerase from linearized cDNA using the mMessage Machine kit (Ambion).

### 5.3 Blue Native Gels and Electrophysiology

Approximately 2–5 ng of cRNA of nvpLGICs were injected into *Xenopus laevis* oocytes of stages V and VI. Injected oocytes were incubated at 19 °C in oocyte Ringer's solution (OR‐2) containing (in mM): 82.5 NaCl, 2.5 KCl, 1.0 Na_2_HPO_4_,1.0 MgCl_2_, 1.0 CaCl_2_, 5.0 HEPES, 0.5 g l^−1^ PVP, 1000 U l^−1^ penicillin, and 10 mg l^−1^ streptomycin. pH was adjusted to 7.3 using NaOH.

For blue‐native gels, His‐tagged nvpLGIC‐1‐ nvpLGIC‐3, nvpLGIC‐5, and human GABA_A_ β3 were expressed in oocytes, purified and resolved by SDS‐PAGE and blue native (BN) PAGE as described [32, 74]. In brief, defolliculated, cRNA‐injected *X. laevis* oocytes were metabolically labelled with [^35^S]methionine, and, after 2 days, surface‐labelled with the membrane‐impermeant dye IR800 NHS ester (LI‐COR) for 1 h. After digitonin solubilization, receptors were purified via their C‐terminal His tag using Ni‐NTA‐Sepharose and resolved by BN‐PAGE in their native and partially LiDS‐denatured state to visualize their oligomeric state. IR800 fluorescence was detected on the wet BN‐PAGE gel using a LI‐COR Odyssey scanner, while ^35^S was detected after drying of the same gel using phosphorimaging. Protein expression was displayed using Image Lab (Bio‐Rad).

For electrophysiology, whole‐oocyte currents were measured two days after injection using a two‐electrode voltage clamp (TEVC) set‐up with a TurboTec 03X amplifier (npi electronic GmbH, Tamm, Germany) and a pump‐driven solution exchange system combined with the oocyte testing carousel (OTC), controlled by the interface OTC‐20. For nvGluCl‐1, the His‐tagged version was functionally characterized. Standard bath solution contained (in mm): 140 NaCl, 10 HEPES, 1.8 CaCl_2_, and 1.0 MgCl_2_, pH was adjusted to 7.4 using NaOH. Solution exchange was controlled using the CellWorks 5.1.1 software (npi electronic). Data were sampled at 0.1–1 kHz and filtered at 20 Hz. Holding potential was −70 mV.

### 5.4 Determination of Surface Expression

Oocytes were injected with ∼20 ng cRNA of nvpLGIC‐2 wildtype or mutants at position K229, carrying an N‐terminal His‐tag. Surface expression was determined as described previously [75]. Briefly, oocytes expressing nvpLGIC‐2 were placed for 30 min in ND96 with 1% bovine serum albumin (BSA) to block unspecific binding, incubated for 60 min with anti‐His rat monoclonal antibody (#3D5, 1:2000; 0.5 µg ml^−1^), washed extensively with ND96/1% BSA, and incubated for 60 min with anti‐rat horseradish peroxidase‐coupled secondary antibody (AP136, 1:10000; Millipore). Oocytes were washed six times with ND96/1% BSA and three times with ND96 without BSA. All steps were performed on ice. Oocytes were then placed individually in wells of microplates, and luminescence was quantified in a Berthold Orion II luminometer (Berthold Detection Systems; Pforzheim, Germany). The chemiluminescent substrates (SuperSignal West Atto, Thermo Scientific) were automatically added, and luminescence was measured after 2 s for 5 s. Relative light units/s were calculated as a measure of surface‐expressed channels. The results are from two independent experiments with oocytes from two different frogs. 8–16 oocytes were analyzed for each experiment and each condition.

### 5.5 Homology Modeling and Molecular Docking

The homology model of pLGIC‐2 in a desensitized state was designed using the SWISS‐MODEL server [76] using the crystal structure of the human β3 homopentamer (PDB ID: 4COF) [33]. The pLGIC‐2 monomer was aligned and superimposed onto each of the five monomers of the homopentameric model using PyMOL (The PyMOL Molecular Graphics System, Version 2.5.4, Schrödinger, LLC), resulting in a complete pLGIC‐2 homopentamer model superimposed onto the 4COF template. The alignment resulted in a root mean square deviation of 0.16 Å between the Cα atoms of the modeled chain and the template.

A model of the GABA‐pLGIC‐2 liganded receptor was studied using molecular docking simulations. Receptor and ligand structures were prepared for docking using the open‐source AutoDock Tools 1.5.7 [77] and binding affinities and optimal ligand‐receptor binding poses were predicted using AutoDock Vina [78]. For this calculation, a 60 × 56 × 56 Å^3^ box was constructed around the GABA‐binding pocket, approximately centered on the Cα atom of F93. The algorithm's exhaustiveness was set to 8, with an energy range of 4. Molecular interactions between the receptor and ligands were analysed using LIGPLOT [79], and the molecular docking results were visualized with Chimera X 1.7.1 [80] to inspect their interaction in detail.

The simulations were prepared using the CHARMM‐GUI Membrane Builder webserver to embed the model and the docked ligand in a 100% POPC membrane, solvated with TIP3P water, and neutralized with 0.15 m NaCl in a 16 × 16 × 16 nm^3^ simulation box at T = 303.15 K. Protein and ligand parameters were generated using the CHARMM36m force field. The K229T mutation was introduced using the CHARMM‐GUI interface.

All simulations were performed with GROMACS 2024.4 patched with PLUMED v2.9.3 [81, 82]. During the initial equilibration steps following the standard CHARMM‐GUI protocol, positional restraints were applied to the protein and lipid heavy atoms for the first six equilibration stages. During the first 50 ns of the production simulations, an adiabatic bias via the ABMD module in PLUMED was applied (kappa = 100 kJ/mol) to stabilize the distance between selected donor‐acceptor pairs (as indicated in Figure 5b). After this initial 50‐ns phase, the simulations proceeded without any bias or restraints. Distances were monitored every 200 ps, and simulation frames were saved every 100 ps.

Clustering analysis of the unbiased section (50 ns to 200 ns) of the second wild‐type replica was performed using the GROMOS method in GROMACS. A root mean squared deviation (RMSD) cutoff of 0.18 nm was chosen to group at least 70% of frames within the first three clusters. The clustering included all protein atoms within 1 nm of the ligand as well as the 15 closest water molecules.

### 5.6 Single Cell Analysis

Expression profiles of all genes of interest were examined across cell state clusters described in [18]. The published dataset was imported to Seurat Vs.4, and expression profiles were visualized with the Seurat::DotPlot function. Cell‐states with above‐average expression in at least 20% of the cells were deemed positive for expression of the putative receptor genes. Putative iGluR genes were identified from the *Nematostella* genome based on Interproscan analysis. All genes annotated with IPR015583 (ionotropic glutamate receptor) were selected and used in downstream analysis.

### 5.7 Fluorescent and Colorimetric In Situ Hybridizations

For whole‐mount in situ hybridization (ISH), embryos were fixed 0.25% glutaraldehyde/3.7% formaldehyde/ PTW (PBS + 0.1% Tween), then in 3.7% formaldehyde/PTW for 1 hr at 4°C. Fixed embryos were washed 4x with PTW and stored in methanol at −20°C. Colorimetric ISH was performed as previously described [83] with minor adaptions: samples were incubated in anti‐Digoxigenin‐AP Fab fragments (Roche) diluted 1:4000 in 0.5% blocking reagent (Roche)/1x MAB (Roche) ON at 4 °C. After 10 × 10 min washes in PTW, samples were rinsed 3 × 5 min with alkaline phosphatase buffer and stained with 1 µL NBT and 1.5 µL BICP in 1 mL alkaline phosphatase buffer as described previously [83]. Embryos were embedded in 86% glycerol and imaged with a Nikon 80i compound microscope equipped with a Nikon DS‐Fi1 camera.

Fluorescent ISH was performed according to Tourniere et al. (Tourniere et al. 2020) with minor adaptations in antibody incubation: Samples were blocked in 0.5% blocking reagent (Roche)/Maleic acid buffer (Roche) for 1 hr at room temperature. Anti‐Digoxigenin‐POD Fab fragments (Roche, 11633716001) and anti‐fluorescein‐POD Fab fragments (Roche, 11426346910) were diluted 0.15 U ml^−1^ in blocking solution. After overnight incubation, samples were washed 10 × 10 min in TNT (0.1 M Tris‐HCl, pH 7.5/0.15 m NaCl/0.5% Triton X‐100), and then incubated in fluorophore tyramide amplification reagent (TSA Plus Kit, PerkinElmer) for 30 min at room temperature. Following the staining reaction, samples were washed with TNT. For double staining, samples were incubated in 2% H_2_O_2_/TNT for 20 min at room temperature after TNT washes and then incubated again in blocking solution before adding the second antibody. Finaly, samples were mounted in VECTASHIELD antifade mounting medium (Vector Laboratories) and imaged with a Leica STELLARIS 5 confocal microscope. Maximum intensity projections were generated using Fiji [84].

### 5.8 Statistical Analysis

*Xenopus* oocytes were randomly assigned to experimental groups without blinding of the experimenter. For electrophysiological experiments, we used cells from a minimum of two batches of oocytes isolated on different days from distinct animals. Each oocyte served as a biological replicate for electrophysiology.

Electrophysiological data were analyzed offline, and current amplitudes were assessed using CellWorks Reader 6.2.2 (npi electronic GmbH, Tamm, Germany). Concentration‐response relationships were normalized to the currents obtained with a saturating concentration (*I/I_max_*). The mean of normalized amplitudes was plotted as a function of ligand concentrations. EC_50_ values and Hill coefficients H were calculated by fitting the data to the Hill equation:

(1) $$\frac{I}{I_{\max}}={\left[1+\left(\frac{\mathrm{EC}50}{A}\right)\right]}^{H}$$

using IGOR Pro (version 8.04, waveMetrics, Inc.), where *I* is the current amplitude, *A* is the concentration of the ligand, EC50 is the concentration of the ligand that produces half‐maximal effect, and *H* is the Hill coefficient.

Desensitization kinetics and recovery from desensitization were fitted to a mono‐exponential function:

(2) $$y=y_{o}+Ae^{\frac{-x}{\tau }}$$

where y_o_ is the offset, A is the amplitude, and τ is the time constant. A fit with a double exponential function did not yield substantially different results, because > 90% of the current decline was described by one component, which was similar to the time constant obtained with a single exponential fit.

To calculate the reversal potential, mean current amplitudes were plotted as a function of voltage and were fitted using a linear equation. The voltage at which the direction of current reversed from negative to positive was considered the reversal potential. Data are presented as mean ± standard deviation. Statistical significance was assessed with one‐way ANOVA followed by a Tukey test, using the software Prism (GraphPad, Boston, USA).

## Author Contributions

A.O. and S.J. performed the functional characterization by two‐electrode voltage clamp; M.B. and S.A. performed homology modelling, A.O.R. performed molecular docking and visualized the model, and S.A. performed molecular dynamics simulation; M.R. performed blue native PAGE; A.G.C. and L.K. carried out and analyzed single cell RNAseq analyses; J.M. performed the phylogenetic analyses; S.K. and L.H. cloned nvpLGIC1‐7; L.K. performed in situ hybridization; U.T. and S.G. designed the study; G.S., U.T., and S.G. supervised the study; S.G. wrote the original draft and all other authors reviewed and edited the original draft.

## Funding

This work was supported by grants of the Deutsche Forschungsgemeinschaft to S.G. (GR1771/8‐1) and to G.S. (Schm536/12‐1), and a grant of the Austrian Science Fund FWF (P34970) and an ERC advanced grant EVONeuroMuscle (101142107) to U.T.

## Conflicts of Interest

The authors have no conflicts of interest to declare.

## Supporting information




**Supporting File**: advs75494‐sup‐0001‐SuppMat.docx.

## Data Availability

Datasets generated and analyzed during this study are included in this published article. Single‐cell data is available from the UCSC Cell browser at “sea‐anemone‐atlas.cells.ucsc.edu”. Code for generating the single‐cell expression plots is available on the Technau Lab GitHub (https://github.com/technau/Cnidarian_pLGIC). Additional information is available from the corresponding authors upon reasonable request.
