# Supplementary material for: Functional Analysis of Ligand‐Gated Chloride Channels in a Cnidarian Sheds Light on the Evolution of Inhibitory Signaling
Source: Adv Sci (Weinh). 2026 May 1;13(41):e15481. doi: 10.1002/advs.202515481 (PMC13335744; doi:10.1002/advs.202515481)
Supplement: Supplementary file 1 — Supporting File: advs75494‐sup‐0001‐SuppMat.docx. [file ADVS-13-e15481-s001.docx]

Supporting Information

Functional Analysis of Ligand-gated Chloride Channels in a Cnidarian Sheds Light on the Evolution of Inhibitory Signaling

Abhilasha Ojha, Linda Kloss, Juan D. Montenegro, Simone Albani, Audrey Ortega-Ramírez, Mihaela Raycheva, Sylvia Joussen, Sabrina Kaul, Michèle Bachmann, Lisa Huf, Günther Schmalzing, Alison G. Cole*, Ulrich Technau*, Stefan Gründer*

**This Supplement includes:**

Supporting Figures S1 to S6


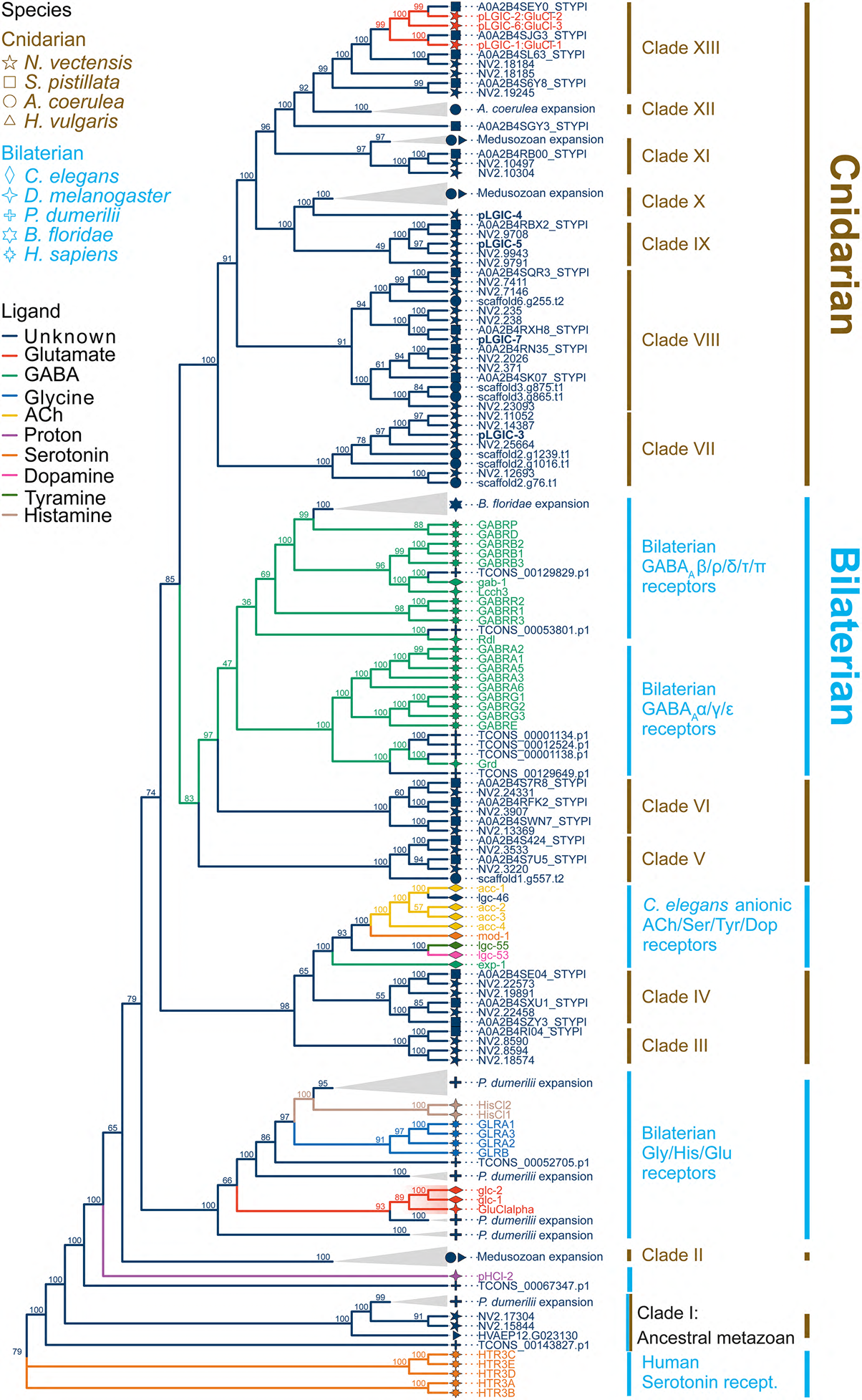


**Figure S1.** Phylogenetic analysis of cnidarian GABA_A_Rs/GlyRs. Maximum likelihood analysis of pLGIC genes in four cnidarians and five bilaterians. The tree is an extension of the tree shown in Figure 2. Cloned receptors of this study are shown in bold. Support (bootstrap) values for individual nodes are indicated. The tree was rooted with mammalian 5-HT_3_Rs as an outgroup.

**Figure S2.** Expression profiles of all putative GABA_A_R-like receptors on the single cell dataset from Cole et al. (2024).^[1]^ Only gene models with at least three reads in the dataset are shown. Most models are expressed at low levels and are concentrated primarily within the mature cnidocytes and class N2 neurons (“neurogland.all.N2”).


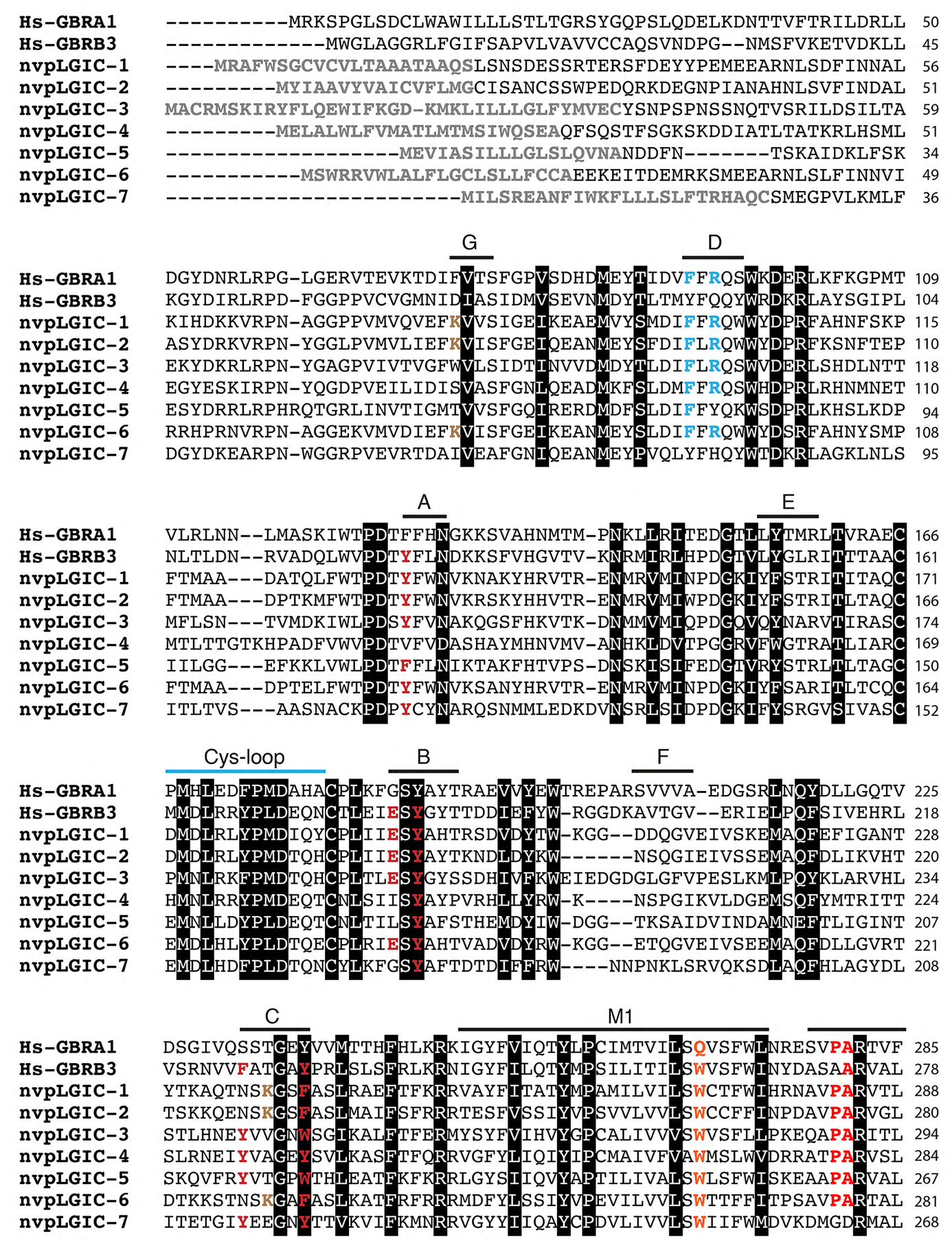


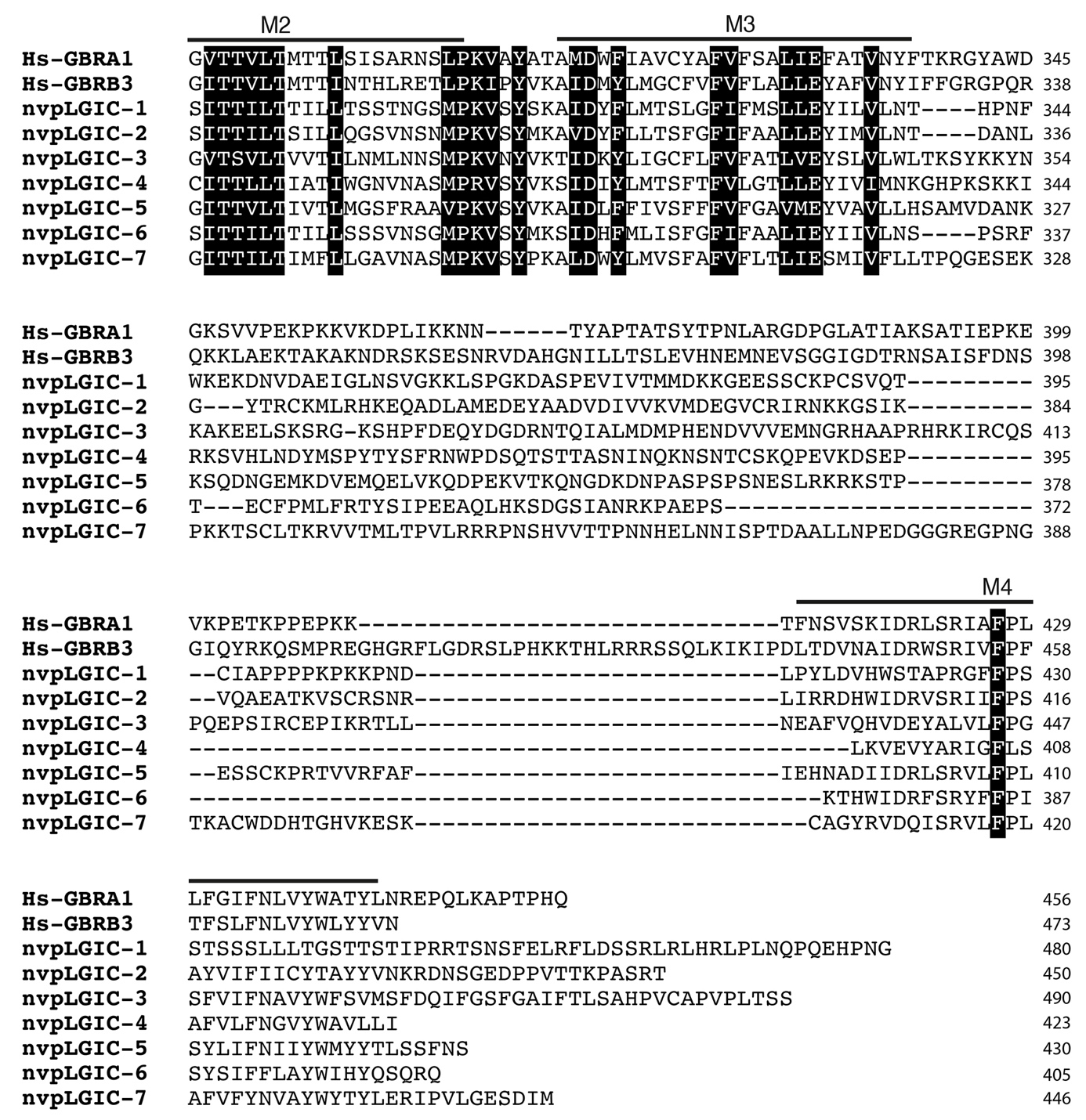


**Figure S3.** Sequence alignment of nvpLGICs with human GABA_A_ α1 and β3. Conserved residues are indicated by white letters on black background. Residues important for ligand binding, ion selectivity and assembly as homomers are indicated by colored letters. Bars represent loops A-G and the transmembrane helices M1-M4; the blue bar represents the Cys-loop. The predicted signal peptides of nvpLGICs are shown in grey; they have been predicted using SignalP 6.0.^[2]^


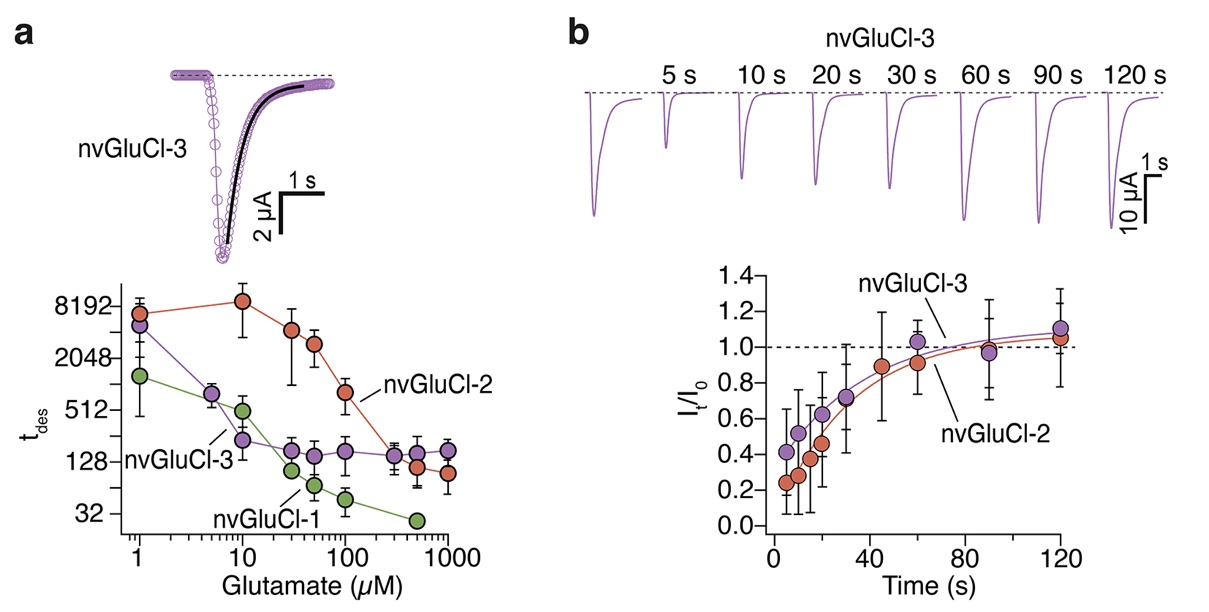


**Figure S4.** a) Top, representative current trace of nvGluCl-3 currents evoked by 100 µM glutamate. Current decline was fitted with a mono-exponential function (black line) to estimate τ_des_. Bottom, mean τ_des_ at increasing concentrations of glutamate for nvGluCl-1 (green), nvGluCl-2 (orange), and nvGluCl-3 (violet), respectively. b) Top, representative current traces showing recovery of nvGluCl-3 from desensitization induced by application of 30 µM glutamate for 30 s. Currents after washout of glutamate for 5, 10, 20, 30, 60, 90, and 120 s are shown. Bottom, mean normalized current responses of nvGluCl-2 (orange) and nvGluCl-3 (violet) as a function of time. Lines represent fits to a mono-exponential function. For nvGluCl-2, a 30 s desensitizing pulse of 100 µM glutamate was used. Data represent the mean ± s.d. of 7-22 cells.

**
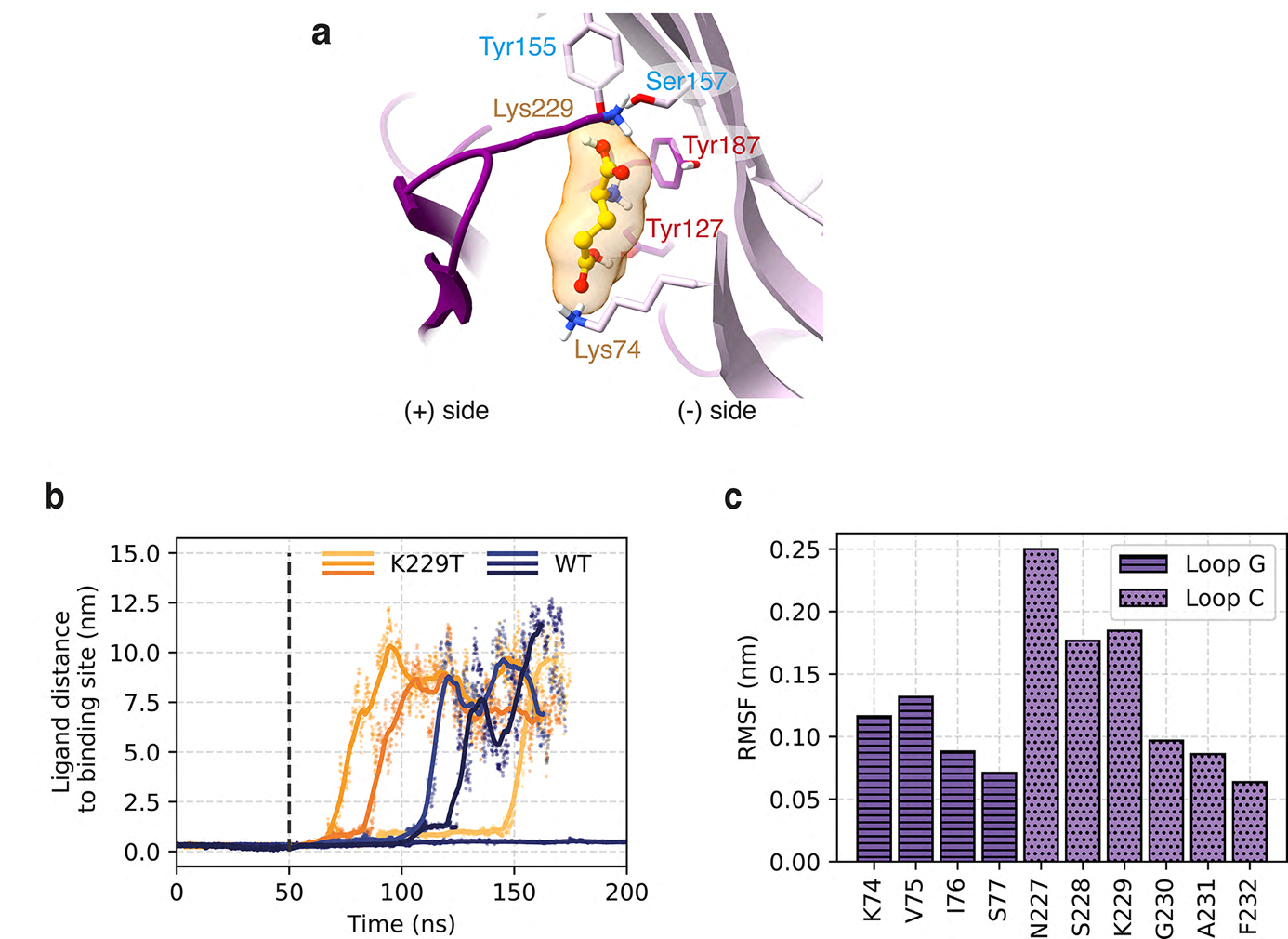
**

**Figure S5.** (a) Docked pose of Glu onto nvGluCl-2. Residue labels of the principal and complementary subunits are shown in red and blue, respectively. K74 and K229 are shown in brown; K74 is from the complementary subunit, and K229 is from the principal subunit. (b) Distances between the ligand and the binding site for the K229T mutant (orange, three replicas) and wild-type nvGluCl-2 (blue, three replicas). The line plot shows the rolling average calculated, using a 10 ns window. Raw data points are displayed as semi-transparent scatter points. The distance was measured between the centers of geometry of the two groups. The simulation segment preceding the vertical black dashed lines corresponds to the phase in which the docking pose was stabilized by an external bias. Upward deflections indicate departure of the ligand from the binding site. (c) Root mean square fluctuation (RMSF) per residue for loop G and loop C of wild-type nvGluCl-2. This value represents the time-averaged positional deviation of each residue from its mean structure during the second replica of the unbiased simulation. Higher RMSF values indicate increased conformational mobility.

**
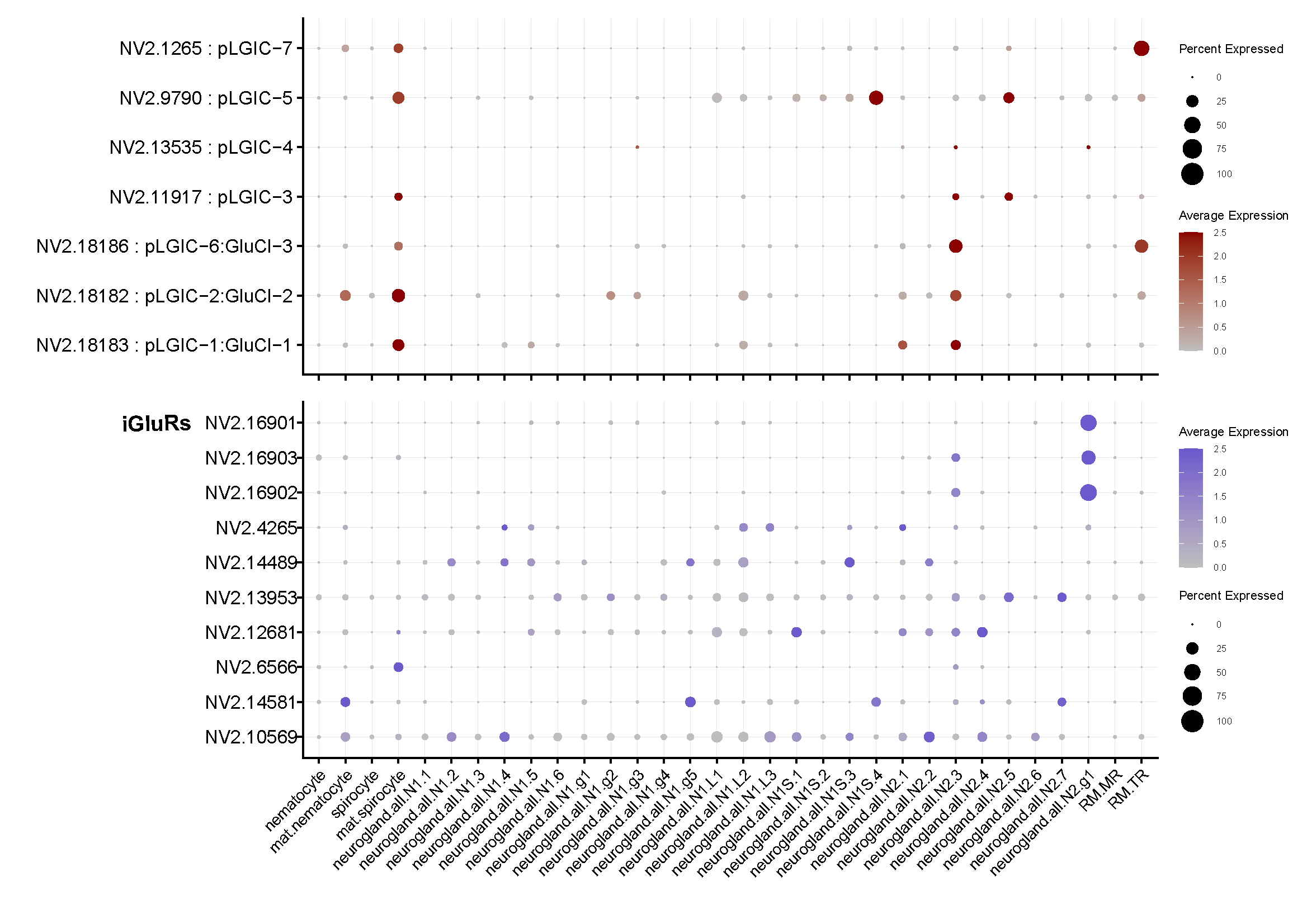
**

**Figure S6.** iGluRs are co-expressed with nvGluCls in a subset of clusters. Expression profiles of selected putative iGluRs (blue dots, bottom panel) and the pLGICs examined in this study (red dots, top panel) across cnidocytes, neurons, secretory cells, and fast muscle in the single cell dataset from Cole et al (2024).^[1]^ Only genes expressed in the selected cell populations are shown. Several iGluRs are co-expressed with one or more nvGluCl, particularly in mature cnidocytes, mature spirocytes and N2.3 neurons. Of note, the high diversity of receptor expression in cluster N2.3 may suggest a heterogeneity within this cluster.

**References**

[1] A. G. Cole, J. Steger, J. Hagauer, A. Denner, P. Ferrer Murguia, P. Knabl, S. Narayanaswamy, B. Wick, J. D. Montenegro, U. Technau, *Front Zool* **2024**, *21*, 8. <https://doi.org/10.1186/s12983-024-00529-z>.

[2] F. Teufel, J. J. Almagro Armenteros, A. R. Johansen, M. H. Gislason, S. I. Pihl, K. D. Tsirigos, O. Winther, S. Brunak, G. von Heijne, H. Nielsen, *Nat Biotechnol* **2022**, *40*, 1023-1025. <https://doi.org/10.1038/s41587-021-01156-3>.
